# Helicobacter Pylori's Plasticity Zones Are Novel Transposable Elements

**DOI:** 10.1371/journal.pone.0006859

**Published:** 2009-09-03

**Authors:** Dangeruta Kersulyte, WooKon Lee, Dharmalingam Subramaniam, Shrikant Anant, Phabiola Herrera, Lilia Cabrera, Jacqueline Balqui, Orsolya Barabas, Awdhesh Kalia, Robert H. Gilman, Douglas E. Berg

**Affiliations:** 1 Department of Molecular Microbiology, Washington University School of Medicine, St. Louis, Missouri, United States of America; 2 Division of Gastroenterology, Department of Medicine, Washington University School of Medicine, St. Louis, Missouri, United States of America; 3 Laboratorios de Investigacion y Desarrollo, Facultad de Ciencias, Universidad Peruana Cayetano Heredia, Lima, Peru; 4 Asociacion Benefica PRISMA, Lima, Peru; 5 Laboratory of Molecular Biology, National Institute of Digestive and Kidney Diseases, National Institute of Health, Bethesda, Maryland, United States of America; 6 Department of Biology, University of Louisville, Louisville, Kentucky, United States of America; 7 Department of International Health, The Johns Hopkins Bloomberg School of Public Health, Baltimore, Maryland, United States of America; 8 Departments of Genetics and Medicine, Washington University School of Medicine, St. Louis, Missouri, United States of America; Universita di Sassari, Italy

## Abstract

**Background:**

Genes present in only certain strains of a bacterial species can strongly affect cellular phenotypes and evolutionary potentials. One segment that seemed particularly rich in strain-specific genes was found by comparing the first two sequenced *Helicobacter pylori* genomes (strains 26695 and J99) and was named a “plasticity zone”.

**Principal Findings:**

We studied the nature and evolution of plasticity zones by sequencing them in five more *Helicobacter* strains, determining their locations in additional strains, and identifying them in recently released genome sequences. They occurred as discrete units, inserted at numerous chromosomal sites, and were usually flanked by direct repeats of 5′AAGAATG, a sequence generally also present in one copy at unoccupied sites in other strains. This showed that plasticity zones are transposable elements, to be called TnPZs. Each full length TnPZ contained a cluster of type IV protein secretion genes (*tfs3*), a tyrosine recombinase family gene (“*xerT*”), and a large (≥2800 codon) orf encoding a protein with helicase and DNA methylase domains, plus additional orfs with no homology to genes of known function. Several TnPZ types were found that differed in gene arrangement or DNA sequence. Our analysis also indicated that the first-identified plasticity zones (in strains 26695 and J99) are complex mosaics of TnPZ remnants, formed by multiple TnPZ insertions, and spontaneous and transposable element mediated deletions. Tests using laboratory-generated deletions showed that TnPZs are not essential for viability, but identified one TnPZ that contributed quantitatively to bacterial growth during mouse infection and another that affected synthesis of proinflammatory cytokines in cell culture.

**Conclusions:**

We propose that plasticity zone genes are contained in conjugative transposons (TnPZs) or remnants of them, that TnPZ insertion is mediated by XerT recombinase, and that some TnPZ genes affect bacterial phenotypes and fitness.

## Introduction

Sets of genes found only in certain strains of a bacterial species are of special interest because their gain or loss can change bacterial phenotypes and evolutionary potentials in ways not typically achievable by point mutation alone [1]. Some of these “strain-specific genes” are in mobile DNA elements, such as transposons, which can insert into genomes without need for the extensive DNA homology required for classical generalized recombination; homology-independent insertion facilitates transposon spread among bacterial species [2]–[4]. Antibiotic resistance determinants are the best known of auxiliary genes in transposons, but determinants affecting other aspects of bacterial phenotype, including virulence in cases of pathogens, are also well documented. Transposable elements attract additional interest because they often alter the expression of genes near their sites of insertion, and generate deletions and other genome rearrangements. They are diverse phylogenetically, in the chemistries used for DNA recognition, cleavage and joining reactions, the involvement of DNA replication in the transposition process, and in how these processes are regulated [2]–[7].

It is with this background that we began studying the “plasticity zones” [8], [9] of *Helicobacter pylori*. This Gram-negative bacterium chronically infects the gastric mucosa of billions of people worldwide, and is implicated in gastritis, peptic ulcer disease and gastric cancer, although most infections are benign, and some are postulated to be beneficial [10]–[12]. This great range in infection outcomes likely reflects multiple host, environmental and bacterial factors. Important in this context is *H. pylori*'s great genetic diversity: any two independent clinical isolates are usually readily distinguished by DNA fingerprinting or sequencing of one or two housekeeping genes; strains also differ in types of virulence genes that they carry; and different genotype clusters predominate in different parts of the world (e.g., East Asia *vs.* Western Europe) [13]–[15]. Prophages seem to be rare or absent, whereas members of the distinctive IS*605* transposable element family are common in *H. pylori* populations worldwide [16], [17]

Superimposed on *H. pylori*'s genome-wide diversity, the segments between the *ftsZ* gene and the 5S,23S rRNA gene pair in the first two *H. pylori* genomes to be sequenced (strains 26695 and J99) [8], [18] were unusually divergent in gene content and arrangement. Only four of the reported 35 orfs reported in this segment of 45 kb from strain J99 were closely related to any of the 55 orfs in the corresponding 65 kb segment from strain 26695. In addition, in strain 26695 this segment had been split in two by a large chromosomal inversion. Based on these observations, the region was named a “plasticity zone”, to connote great genetic variability. Its G+C content was lower (34–35%) than that of the *H. pylori* chromosome overall (39%), which suggested horizontal transfer from unrelated bacterial species. It was in the same chromosomal location in both strains (after correction for 26695's large inversion), and therefore gave no indication of transposition.

Several plasticity zone orfs were implicated by protein level homologies variously in specialized recombination (a tyrosine recombinase family member) [19] (here designated “*xerT*”; equivalent to genes in GenBank accessions referred to variously as *xerCD*, *xerC* or *xerD*), the regulation of DNA supercoiling and gene expression (*topA*, DNA topoisomerase) [20], [21], and DNA separation at cell division or preparation for DNA transfer by conjugation (*parA*) [22], [23]. We had also found a 16 kb gene cluster that encodes a type IV protein secretion complex, called *tfs3*, that was embedded in some plasticity zones [24]. This was the third type IV secretion system gene cluster found in *H. pylori* and is distinct from the two others: (i) a gene cluster in the *cag* pathogenicity island (*cag PAI*) whose encoded proteins mediate delivery of CagA protein to mammalian cells, where CagA affects parameters such as cytoskeletal and tissue structure, cell proliferation and apoptosis; and that also mediate delivery of peptidoglycan fragments that induce proinflammatory cytokine synthesis [25], [26]; and (ii) a second gene cluster that confers competence for DNA transformation (*comB* locus) [27]. Much of the *tfs3* gene cluster is present in strain J99 [24], although it was not included in the report of this strain's genome sequence [8]; a *tfs3* fragment is also present in strain 26695's plasticity zone [24]. *tfs3*'s function is not yet known, but possibilities include a role in bacterial conjugation, or host cell signaling complementary to that of the *cag PAI-*encoded system.

Several plasticity zone genes with no homologs of known function had been associated epidemiologically with overt disease or more benign infection in certain human populations; many are transcribed during growth in culture [28]–[30]; and one plasticity zone encoded protein, JHP0940, was produced in *E. coli* carrying the cloned gene, and was found to stimulate synthesis of the eukaryotic transcription regulatory factor NFkappaB when added to cultured mammalian cells [31]. Collectively, these findings encouraged thinking that many plasticity zone genes are functional, and that some could affect *H. pylori* phenotypes such as persistence or virulence in particular host environments.

Here we sought to better understand the nature of plasticity zones, and how they are acquired and evolve in bacterial populations. Our results indicate that plasticity zones are novel transposons (TnPZs); that TnPZs are of only modest genetic diversity and can be relatively stable; and that the reference strains 26695 and J99 contain mosaics of several TnPZ remnants that collectively encompass much of the diversity found in *H. pylori's* various intact TnPZ elements.

## Results

### Plasticity zone genes are common in *H. pylori* strains worldwide

An initial PCR-based survey of the distribution in *H. pylori* populations of 16 representative plasticity zone orfs (11, three, one and one from strains J99, 26695, PeCan18B [24, GenBank Accession AF487344] and CPY6081 [24, GenBank Accession AY128680], respectively) identified an average of six orfs per strain in 94 of 102 strains screened, variously from Spain, Japan, India, Peru, and Gambia (Tables S1A, S2). None of these orfs were found in the other eight strains, suggesting that those strains might be plasticity zone-free. In accord with this, no plasticity zone orfs were found in the published genome sequences of *H. pylori* strain HpAG1 and of a strain of *H. acinonychis* (gastric pathogen of big cats; closely related to human *H. pylori*) [32], [33; Genbank Accessions NC_008086 and NC_008229]. In addition, the apparent abundance of certain genes varied markedly between populations. For example, gene *jhp0947* was found in eight of ten Gambian strains, but not in any of 22 Japanese strains, whereas *hp0441* and *hp0446* were found more frequently in strains from Japan (15 of 22) than from elsewhere (*jhp* and *hp* refer to genes found in reference strains J99 and 26695, respectively). *jhp0940*, whose product had been implicated in inflammatory responses to infection and virulence [31], was found in just eight of these 102 strains. Equivalent diversity in plasticity zone gene content had also been found by DNA hybridization in Costa Rican and Mexican strain collections [28], [29].

### Plasticity zones as novel transposable elements

We sequenced five plasticity zones: four from strains of *H. pylori*, and one from a strain of *Helicobacter cetorum* (a distinct *Helicobacter* species from a Beluga whale); identified their locations in other *H. pylori* strains; and analyzed them in two additional complete *H. pylori* genome sequences that were released during preparation of this manuscript (strains G27, and P12, GenBank Accessions CP001173 and CP001217).

**Figure 1:**
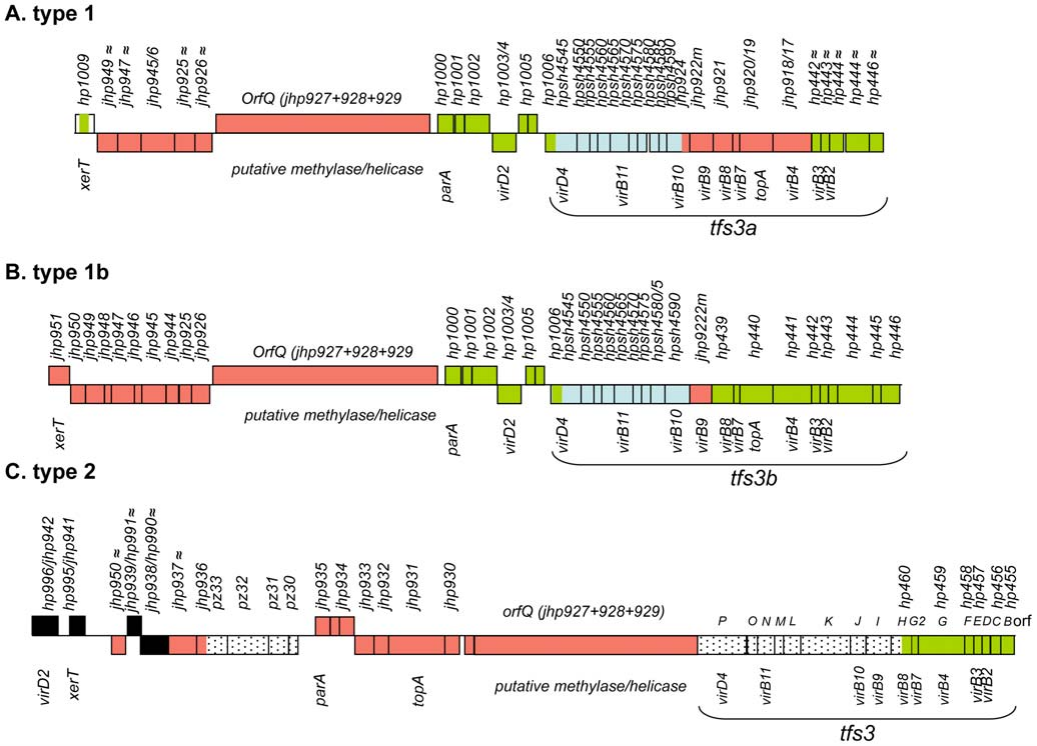
Three types of TnPZs. Coding: boxes in green, orfs with homologies to genes in reference strain 26695 (orf numbers start with *hp*); in red, orfs with homologies to reference strain J99 (orf numbers start with *jhp*); in black, orfs common to strains 26695 and J99; boxes in blue (*hpsh_04545 - hpsh_04590*), first found in Shimaa strain Shi470 and then in other type 1 and type 1b TnPZs (Figs. S1, S2, S6); boxes with dots, orfs first found in strain PeCan18B and then in all full size type 2 TnPZs (*tfs3 orfs H-P*, and orfs *pz30-pz33*); Orfs marked with ≈, relatively low protein-level identities to their homologs in reference strains 26695 and J99 (range of 35–70%); other orfs that are not marked with ≈ have protein identities of 90–95% to those in these reference strains. Orfs transcribed rightward are represented above the main line, and those transcribed leftward are shown below the line. Note that adjacent orfs in Shi470 were numbered in fives (*hpsh_04545, hpsh_04550, hpsh_04555*, etc.) by the NCBI genome annotation pipeline team. Designations B through Q correspond to orf names used in our initial description of the *tfs3* type IV secretion gene cluster [24]. A. Type 1 TnPZ. This type was found in the strain Shi470 genome sequence (GenBank Accession CP001072, *hpsh_04480-hpsh_04640*), in 35 of the other 44 Shimaa village strains (by PCR), and also in the strain G27 genome sequence ([34], GenBank Accession CP001173, *HPG27_986 - HPG27_959*). These TnPZs have type 1 specific *xerT* genes (full length versions of strain 26695's truncated *hp1009* gene) on the left, a *tfs3a* element on the right, and a centrally located type 1-specific *virD2* allele. Although most genes are closely related to genes found in reference strains 26695 and J99, some of them (*jhp0949*, *jhp0947*, *jhp0925-6* near the left end and *hp0442 – hp0446* near the right end, marked with ≈) are divergent allelic forms, specific for type 1 TnPZ elements. B. Type 1b TnPZ, as in strain P12 (GenBank Accession CP001217, orfs *HPP12_437 - HPP12_473*), is likely to be a hybrid element, consisting of a type 1 TnPZ central region (*orfQ* through *virB9*) that contains ∼8 kb segments at each end from another distinct TnPZ element, unrelated at the DNA sequence level, although similar in gene content and arrangement, to type 1 TnPZs. Type 1b-specific sequences are present in 26695 and J99 plasticity zones. C. Type 2 TnPZ, found in *H. pylori* strains PeCan18B (GenBank Accession AF487344), Shi170 (GenBank Accession EU807988) and P12 (GenBank Accession CP001217, orfs *HPP12_01320- HPP12_01353*), and also in a strain of *H. cetorum* (GenBank Accession EU015081). Type 2 TnPZs start with type 2 specific *virD2* and *xerT* genes on the left and end with the *tfs3* gene cluster on the right. The region between *jhp0939/hp0991* and *xerCD* (2.4 kb) is variable among type 2 strains.

**Figure 2:**
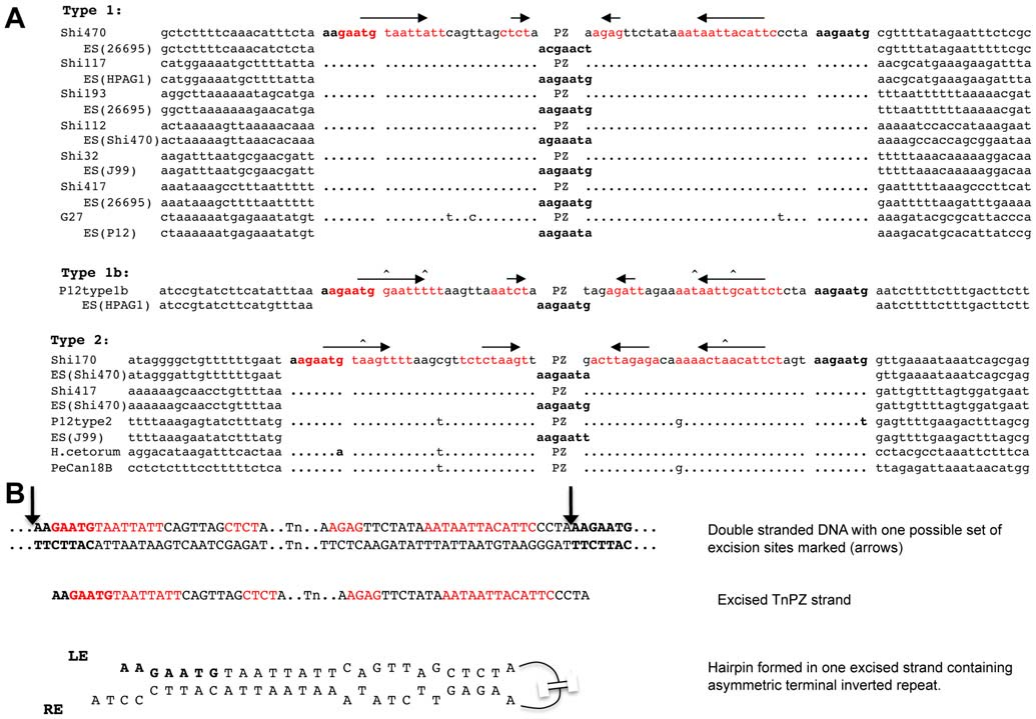
TnPZ flanking sequences, termini, insertion sites and corresponding empty sites. A. Alignments of sequences at multiple sites of insertion. A seven bp target (5′AAGAATG or closely related sequence) is present as direct repeats at each TnPZ end, and in single copy at unoccupied (empty) sites. Inverted repeats that could form foldback (hairpin) structures in single stranded DNA are marked in red and underlined, with their orientation shown by arrows. Corresponding empty site (ES) locations were chosen from strains with the highest sequence matches. TnPZs and remnants from strains 26695, J99 and HUP-B43 were not included due to their chimeric nature and rearrangements. The sequence from strain PeCan18B includes only its type 2 TnPZ (type 1b remnant is missing several hundred bp at its normal TnPZ end, including its 5′AAGAATG target). No corresponding empty site for PeCan18B was found; this we ascribe to the variable and repetitive nature of sequences in the 5S,23S rRNA *- ftsZ* interval (Fig. S9). Type 1 TnPZ insertion sites: Shi470 and five other Shimaa strains, in *hp0488*; Shi117, in gene *HPAG1_0627* (*frxA*, encodes NAD(P)H-flavin oxidoreductase); Shi193 and 17 other Shimaa strains, in intergenic region between *hp0209* and *hp0210*; Shi112 and eight other Shimaa strains, in intergenic region between HPSH_07175 and the rRNA gene *HPSH_r08356* (equivalent to *jhp1299* – *5S,23S rRNA* interval of J99); strain Shi32, in jhp0244 (methyltransferase of type II restriction modification system); strain Shi417, between *hp1159* and *hp1160*; strain G27, between *HPP12_1009* and the tRNA gene *HPP12_t21*. Type 1b TnPZ insertion site: strain P12 in gene *HPAG1_0439* (encodes type I restriction-modification R protein (hsdR)). Type 2 TnPZ insertion sites: Shi170 and seven other Shimaa strains, between *HPSH_01160* and *HPSH_01165* (*jhp0210* and *jhp0211* in strain J99); strain Shi417, in *HPSH_07895* (encodes type I restriction modification R protein (hsdR)) of strain Shi470 (equivalent to *jhp1424* of strain J99); strain P12 (type 2), in *jhp1272*; *H.cetorum*, no counterpart for this insertion site in *H. pylori* genome sequences; strain PeCan18B type 2, between the 5S,23S rRNA gene pair and *ftsZ* (*jhp0913*). B. Hairpin structures involving TnPZ termini. This illustrates a hypothetical excision of one TnPZ strand, a possible product of TnPZ transfer from strain Shi470 to recipient cells. The sites of single strand cleavage (arrows) are also placed arbitrarily, to maximize hairpin length and to cut at the ends, rather than within, the heptanucleotide target sequences. The third line illustrates the type of foldback (hairpin structure) that would be formed by annealing of complementary sequences from terminal inverted repeats. An equivalent structure would be formed by excision of the other DNA strand.

The most compelling evidence for transposition emerged in studies of our collection of 44 strains from residents of Shimaa, a Machiguenga village (∼600 residents) in the remote Peruvian Amazon. We had sequenced the genome of one of these strains (Shi470) (GenBank Accession CP001072), and found within it a 39 kb DNA segment (Fig. 1A, Fig. S1), some ∼85% of which was related to sequences assigned to the plasticity zones of strains J99 or 26695. This segment, however, was inserted cleanly into Shi470's homolog of gene *hp0488*, which is far from the 5S,23S rRNA - *ftsZ* region in which strain 26695's and J99's plasticity zones are located. The inserted DNA was flanked by a direct repeat of 5′AAGAATG; one copy of a closely related heptanucleotide was found at the unoccupied *hp0488* site in other unrelated *H. pylori* strains (Fig. 2). This insertion into *hp0488* was ascribed to transposition, and the name “TnPZ” was devised to connote “transposon, plasticity zone”. This TnPZ was further designated “type 1”, as will be explained below.

The finding of a transposon at a new location in Shi470 that contained all plasticity zone genes in this strain's genome led us to ask if this element was stable or highly variable (plastic) in gene content, and if it could also insert at other sites. These issues were pursued using the 43 other Shimaa village strains, in the expectation that studies of transposon dynamics in a small and isolated population would provide valuable insights, not easily obtained with strain collections from more frequently studied cosmopolitan communities.

We started with PCR tests for six DNA segments from strain Shi470's TnPZ (one segment near each TnPZ end, four internal). Each segment was amplified successfully from 35 of the other 43 Shimaa village *H. pylori* strains (primers listed in Table S1B; PCR products same size from each strain), but not from any of the other eight strains. The TnPZ locations in these 35 strains were identified by PCR with primers specific for already identified insertion sites, and by direct chromosomal sequencing from element ends when needed. Five additional TnPZ insertion sites were found (Fig. 2). The inserted DNAs were flanked by direct repeats of 5′AAGAATG, a single copy of which was present at most corresponding empty sites in other strains, and could be in either orientation in the chromosome. The TnPZ ends at each insertion site were constant in sequence for at least 80 bp, and contained 13 bp terminal inverted repeats (Fig. 2). Insertion at multiple positions, short target sequence duplications, and terminal inverted repeats (which typically constitute sites of transposase action) are characteristic of many classes of transposable elements [2], [4], [5].

To test for conservation of gene content and arrangement, PCR was carried out on nine representative Shimaa strains that included at least one from each site of TnPZ insertion, using 12 primer pairs that collectively covered the entire length of Shi470's TnPZ in overlapping ∼4–5 kb long segments (primer pairs in Table S1B). With just two exceptions, each strain yielded products whose sizes matched those from Shi470. The two exceptional strains each contained a deletion of ∼0.5 kb in or near gene *orfQ*; and their TnPZs were each inserted at the same site (between *jhp1299* and a second 5S,23S rRNA gene pair). Further PCR tests of the seven additional strains with TnPZ inserted at this *jhp1299*-linked site identified the same ∼0.5 kb deletion in each of them, suggesting derivation from a recent common ancestor. No other variation in size or gene arrangement in TnPZs inserted at any of the other five chromosomal sites was detected. A 39 kb element with the same gene content and order as that in Shi470, and ∼95% identity to it in DNA sequence, was identified in the recently released genome sequence of Italian strain G27 [34] (GenBank Accession CP001173) (Fig. S2). This TnPZ was inserted at a seventh site, and was also flanked by a 5′AAGAATG target duplication (Fig. 2). These results indicate that type 1 TnPZs are widespread, not unique to our special Amerindian strain collection. The occurrence of closely related TnPZs in unrelated strains from different continents further illustrates TnPZ stability and mobility.

A 52 kb segment, most of whose encoded proteins were homologs of those of the smaller (∼39 kb) type 1 TnPZs was found in the urban Peruvian strain PeCan18B, and was sequenced by chromosome walking from the previously characterized “*tfs3*” type IV secretion gene cluster [24] (GenBank Accession AF487344). This segment differed significantly in gene arrangement and DNA sequence from that in Shi470. Accordingly, this PeCan18B DNA segment was designated “type 2”, for convenience in distinguishing it from “type 1” TnPZs (Fig. 1, Fig. S3). However, this type 2 segment was between the 5S,23S rRNA and *ftsZ* genes, as are the plasticity zones of strains J99 and 26695, and thus its position did not give information as to whether it was or was not mobile.

Type 2 TnPZs were also found by PCR in nine Shimaa village strains. The element in one of them (Shi170) was sequenced (GenBank Accession EU807988) and found to be well matched in gene content, order and DNA sequence (86–90% DNA identity) to PeCan18B's type 2 TnPZ (Fig. S4). However, it was inserted at a different chromosomal site (between homologs of *hp0224* and *hp0226*), thereby indicating that type 2 TnPZs are also transposable.

All nine Shimaa strains that contained a type 2 TnPZ also contained a type 1 TnPZ and belonged to either of two clonal groups. In one group (eight strains, including Shi170) the type 1 TnPZ was inserted between *jhp1299* and a second 5S,23S rRNA gene pair, and the type 2 TnPZ was inserted at the site used in Shi170 (*hp0224* - *hp0226* interval). Each of these strains had the same 0.5 kb deletion in or near *orfQ* and an *orfG*::IS*Hp608* insertion in their type 1 and type 2 TnPZs, respectively. In the second group (one strain) the TnPZs were located at other sites (type 1, between *hp1159* and *hp1160*; type 2, in *hp1402*); this strain lacked the above-mentioned 0.5 kb deletion in or near orfQ and the *orfG*::IS*Hp608* insertion.

The genome sequence of German strain P12 (GenBank Accession CP001217) also contained two different TnPZs. It's type 2 element was 17 kb shorter than that of PeCan18B because of three internal deletions, but was 95%–99% identical to PeCan18B's TnPZ in shared sequences (Fig. S5). This TnPZ variant was located at a fourth site (in *jhp1272*). Strain P12's other element was similar to other type 1 elements in gene organization (Fig. S6), but was unrelated to them in DNA sequence for the first ∼8 kb from each end; this divergence indicates derivation from a separate TnPZ lineage. However, its central part (*orfQ-virB9*) was ∼95% identical in sequence to that of other type 1 elements. These features suggested a recombination event between two types of TnPZs: a type 1 TnPZ and another previously unknown type. The apparently hybrid TnPZ in strain P12 is called type 1b. It was inserted at a unique chromosomal site (in *hp0464*, an *hsdR* gene), was bracketed by a 5′AAGAATG target duplication, and had a terminal 14 bp inverted repeat (Fig. 2).

Finally, a 55 kb type 2-like TnPZ element was found in an isolate of the distinct *Helicobacter* species, *H. cetorum*, following an unexpected success in PCR amplification with three pairs of primers that had been designed for plasticity zone genes of reference *H. pylori* strain J99 (*jhp0931*, *jhp0940*, *jhp0941*). The *H. cetorum* TnPZ differed from *H. pylori* type 2 TnPZs primarily in containing 8.5 kb of extra DNA in six segments, three of which were inframe insertions in the *orfQ* gene (Fig. S7). It was 80–85% identical in shared sequences with *H. pylori* type 2 TnPZs, and was inserted at a distinct chromosomal locus that has no counterpart in current *H. pylori* genome sequences. The ends of type 2 TnPZs from these various sources were well-matched to one another (≥90% identity, for >80 bp), but were unrelated to the termini of type 1 TnPZs. Type 2 TnPZ ends also contained terminal inverted repeats and were flanked by direct repeats of 5′AAGAATG or a closely related target sequence (Fig. 2).

### Vestigial and remnant TnPZs

Although the just-described type 1 and type 2 TnPZs seemed stable in some *Helicobacter* strains, other strains contained deletions and rearrangements of TnPZ sequences. As one example, strain PeCan18B contained a 3 kb remnant of a type 1b TnPZ adjacent to its full-length (49 kb) type 2 TnPZ (Fig. S3). This likely resulted from type 2 TnPZ insertion into a pre-existing type 1b TnPZ, and deletion from one type 2 TnPZ end (Transposable elements are well known to generate adjacent deletions and other rearrangements in transposition-like events [35]–[37]). As a second example, the 27 kb type 1b TnPZ of strain HUP-B43 contained a large internal inversion (genes pz4b-13b), one end of which coincided with an inserted IS*Hp608* element. Deletions, relative to the full length type 1b TnPZ in strain P12, were also seen at each of HUP-B43's inversion breakpoints (see Fig. S8). Although IS*Hp608* was implicated by its position at one breakpoint, the absence of a mobile element at the other breakpoint implicated an additional spontaneous event.

**Figure 3:**
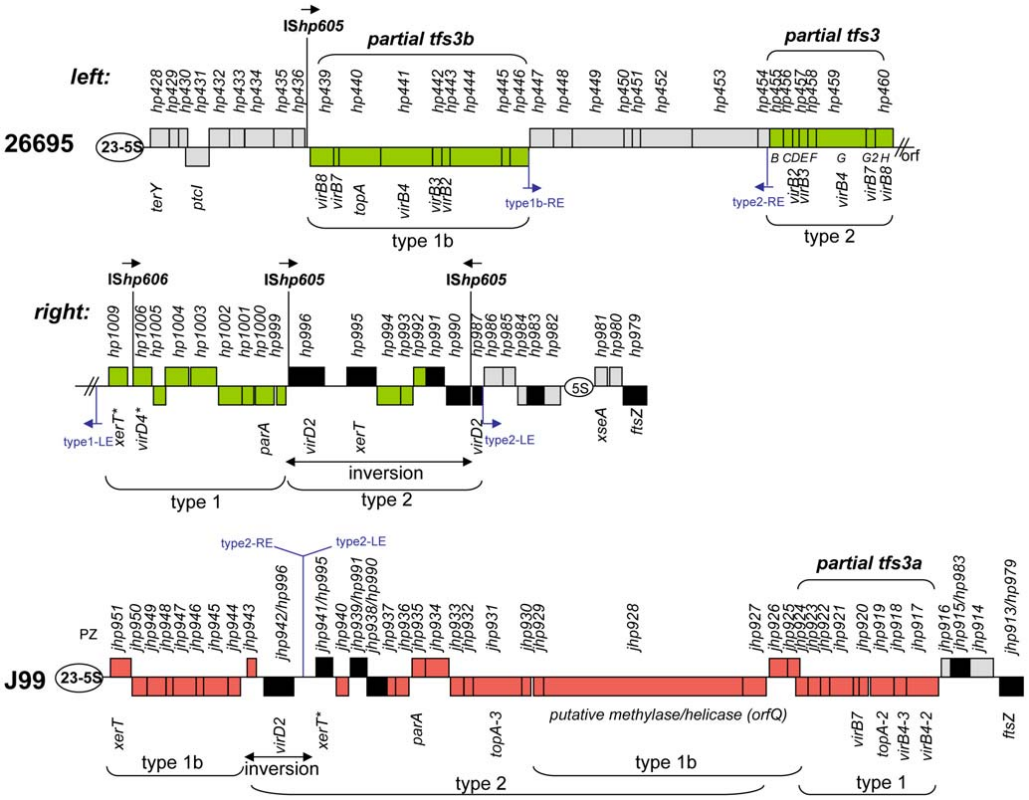
Plasticity zones of reference strains 26695 and J99 ([8], [18], GenBank Accessions AE000511 and AE001439). Color coding: boxes in green, TnPZ orfs found in 26695 but not J99; in red, TnPZ orfs found in J99 but not 26695; in black, orfs found in both strains; in grey, orfs located between *ftsZ* gene and 5S, 23S rRNA gene pair that are judged to be not of TnPZ origin. TnPZ left and right ends are designated LE and RE, with arrows pointing outward. Strain 26695. This segment is inferred to have resulted from multiple TnPZ insertions, and deletions that left only remnants of original full-length elements. It was also split in two by a large chromosomal inversion (breakpoint indicated by //). This breakpoint is associated with a type 1 TnPZ, suggesting that it had mediated the inversion. Strain J99. This plasticity zone (*jhp0917-jhp0951*) is located between the *ftsZ* (*jhp0913*) gene and the 5S,23S rRNA gene pair, and is composed of fragments from type 1 (*jhp0917-jhp0924*), 1b (*jhp0944-jhp0951*; *jhp0925, jhp0926* potentially part of *jhp0927-jhp0929* (*orfQ*) as well); and type 2 (*jhp0943-jhp0930* and potentially part of *orfQ* as well) TnPZs. A lack of clustering of *orfQ* gene sequences according to TnPZ types, however, precluded more precise identification of the boundary between type 1b and type 2 sequences. *jhp0942* and flanking regions (0.9 kb next to 5′-end, 1.15 kb next to 3′-end, which also contains a short *jhp0943* orf), are inverted relative to nearby genes. One inversion breakpoint coincides with juxtaposed left and right ends of a type 2 TnPZ. *jhp0941* (labeled *xerT*) is a composite orf in which the first 114 *xerT* codons have been replaced by 90 codons of *orfB* of *tfs3*. These interpretations are provisional, however, because the J99 genomic DNA that had been provided to the genome sequencing team contained a *tfs3* gene cluster and a second copy of *orfQ* [24], which were not included in the reported genome sequence [8], and the possibility of additional errors has not been assessed.

Far more complex histories of recurrent TnPZ insertion, deletion and rearrangement were identified in the plasticity zones of reference strains 26695 and J99 (Fig. 3), based on the sequence content of the several types of complete TnPZs defined above. In brief, most deletions and rearrangements in reference strain 26695's plasticity zone seemed to be transposable element-mediated. To illustrate, the left half of this zone contained type 1b and type 2 TnPZ fragments separated by sequences that were not found in intact TnPZ elements (*hp0447- hp0454*), but that were present in the highly variable empty site region between the 5S,23S rRNA and *ftsZ* genes of strains P12 and G27 (described below). The type 1b and type 2 remnants each terminated with typical right end sequences and the characteristic heptanucleotide target (5′-AAGAATG), which implies two early TnPZ insertion events. However, the broken ends of the type 1b and type 2 remnants coincided with an inserted IS*605* and the endpoint of the chromosomal inversion that had split the plasticity zone in two, respectively.

The right half of 26695′s split plasticity zone contained remnants of type 1 and type 2 TnPZs that both terminated with characteristic left end sequences. This feature also implied early TnPZ insertion events. The type 1 remnant was inserted at the just-mentioned chromosomal inversion breakpoint. IS*605* marked the broken ends of the neighboring type 1 and type 2 remnants, and a second IS*605* had split the type 2 remnant's *virD2* gene (5′ and 3′ fragments, given gene numbers *hp0987* and *hp0996*; Fig. 3). These two IS*605*s also marked the breakpoints of the type 2 remnant's interstitial inversion. Finally, IS*606* was inserted at the endpoint of a truncated *virD4* gene (*hp1006*). The positions of these IS elements indicated that they had caused the adjacent deletions and inversions of plasticity zone sequences. The position of the type 1 remnant suggested that its ancestral type 1 TnPZ had been responsible both for the large chromosomal inversion and the associated deletion of many genes from this plasticity zone's ancestral type 2 TnPZ.

The plasticity zone in the published genome sequence of strain J99 [8] contains segments from all three types of TnPZ elements. Only one pair of TnPZ end sequences was found - the juxtaposed type 2 right and left ends between *virD2* (*jhp0942*) and *xerT* (*jhp0941*), which coincides with inversion of the *virD2*-containing segment of this plasticity zone (Fig. 3). No IS elements or other TnPZ were found in the J99 plasticity zone sequence, and thus, in contrast to the case of strain 26695, spontaneous deletion events are implicated in generating most novel aspects of this zone's structure.

### *ftsZ*-linked empty sites

Our initial PCR-based *H. pylori* strain survey (Table S2), had included a test for “empty sites” in the 5S,23S rRNA - *ftsZ* interval, which is occupied by the plasticity zones in strains 26695 and J99. Each of 22 Japanese strains yielded a PCR product that ranged from 2 to 4 kb in size, as did a fraction of strains from several other countries (11/22, 13/24 and 4/22 from Spain, Peru and India, respectively, but 0/10 from The Gambia). Success in amplification indicates that TnPZ is not inserted in this interval. There are several explanations for cases of non-amplification: the presence of TnPZ or multiple other (non-transposon) genes; a chromosome rearrangement endpoint in this interval; or sequence divergence in a primer binding site.

The sequences of this interval from eight strains (Fig. S9A) revealed marked variability in size (from 2 to 20 kb) and gene content (Fig. S9B); only one orf (*jhp0915*) was common to each such empty site. Equivalent diversity was seen in this interval next to TnPZs (full length or remnant) in another four strains. These data were used to help distinguish TnPZ remnants from other sequences in strain 26695's complex plasticity zone (Fig. 3), and thereby gain insight into its evolutionary history. Further study is needed to identify the source of this region's variability – perhaps transposable element insertion-related activities, local enhancers of spontaneous mutation, or variable selection pressures.

### Type IV secretion *(tfs3)*

Many type IV secretion systems are used to deliver DNA and proteins during bacterial conjugation [3], [38]. Each type of full-length TnPZ element contained a ∼16 kb long cluster of type IV secretion genes designated *tfs3* (Fig. 1). We had previously described one version of this cluster in the type 2 TnPZ of strain PeCan18B and remnants of it in reference strains 26695 and J99 [24]. The *tfs3* gene clusters of type 2 TnPZs in *H. pylori* strains P12 and Shi170 and in the *H. cetorum* strain shared 99%, 87% and 86% identities, respectively, with that of PeCan18B. A segment encoding equivalent proteins, although highly divergent from canonical *tfs3* in DNA sequence, was present in the type 1 TnPZs of strains Shi470 and G27 (97% DNA identities between them), and will be referred to as *tfs3a*. The corresponding segment in the type 1b TnPZ of strain P12 appeared to be of mixed ancestry: half of it (*virD4* through *virB9*) was closely matched (97%) to corresponding *tfs3a* DNA sequences. The remainder (*virB8* to end) was unrelated in DNA sequence to corresponding *tfs3* or *tfs3a* segments, although it encoded substantially the same proteins. For convenience, this gene cluster will be referred to as *tfs3b*. It is attractive to imagine that *tfs3* encoded complexes mediate conjugational transfer of TnPZ transposons.

### *xerT*, tyrosine recombinase family gene

**Figure 4:**
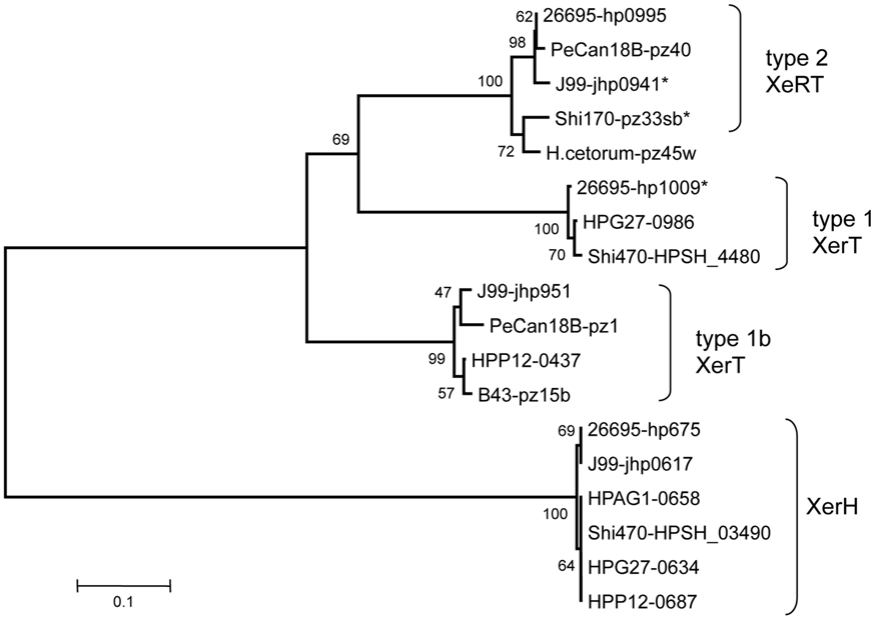
Neighbor-joining tree of *Helicobacter* Xer (tyrosine recombinase) protein sequences with bootstrap values. Most *H. pylori* strains contain two divergent *xer* genes of 356 codons on average, one chromosomal (*xerH*) and one in TnPZ (*xerT*) (protein identity about 26%) [see multiple alignments, Fig. S10). TnPZ *xerT* genes can be subdivided into three allele types that correspond to TnPZ type (1, 1b and 2), with protein identities between them of about 53%. Note that *jhp0941* is a mosaic of 90 codons of tfs3 *orfB* sequence and 241 codons of type 2 *xerT* sequence. Only the 241 codon *xerT* component of *jhp0941* was used in this analysis. The two or more types of TnPZ *xerT* genes found in a few strains reflect the presence of one remnant plus a full length TnPZ (PeCan18B) or of multiple TnPZ remnants (26695, J99) in them. Orfs marked with an asterisk are pseudogenes that have been inactivated by point mutation (frameshift, internal stop), insertion or deletion.

Most naturally occurring transposons encode their own element-specific transposase proteins. The product of the gene designated “*xerT*” in each full length TnPZ (Fig. 1) is a good candidate for its transposase. Each inferred XerT protein exhibits ∼28% amino acid identity to the canonical 298 residue XerC and XerD recombinases of *E. coli* over the carboxy-terminal two-thirds of their 355–363 amino acid lengths, and contains residues that likely correspond to residues Tyr279, Arg148 and Arg 247 that are important in the XerD active site [39]. These features place them in the diverse tyrosine recombinase family, whose members also include phage lambda integrase, Cre recombinase and the transposase of conjugative transposon Tn*916* [40], [41]. The TnPZ encoded XerT proteins fall into three distinct phylogenetic clusters that correlate with TnPZ type (1, 1b and 2), with sequence identities among the types of 55% to 62% (Fig. 4). Each XerT type is associated with particular TnPZ end sequences (Fig. 2). This makes it attractive to imagine XerT-end sequence co-adaptation: e.g., that type 1 XerT protein acts on type 1 TnPZ ends, but not type 2 TnPZ ends, and vice versa. Each *H. pylori* strain also contains a second chromosomal *xerC/xerD* homolog (recently designated *xerH* [42] with only ∼26% amino acid level identity to those in TnPZs. XerH proteins form just one cluster (Fig. 4), in keeping with the idea that they should be specific for a unique “*dif*” site that is well conserved among *H. pylori* strains [19], [42]. Preliminary sequence inspections using PSI-PRED algorithms predict similar secondary structures and perhaps folds for the *H. pylori* and *E. coli* Xer proteins (Fig. S10).

### virD2, parA and topA

Three other TnPZ genes are interesting in terms of the idea that TnPZs undergo conjugative transposition. One, labeled *virD2*, encodes a domain that is characteristic of the VirD2 relaxase and that extends over nearly the first half of the protein's ≥635 amino acid length. Well-characterized VirD2 proteins make a single strand cut at the origin of conjugational DNA transfer and remain bound to the freed 5′ end during its transfer to recipient cells. *Agrobacterium* VirD2 also helps mediate insertion of this end into target DNAs [38], [43]; the corresponding relaxases of transferable plasmids probably mediate recircularization of transferred (linear) plasmid DNA in recipient cells [44]. The TnPZ *virD2* gene, located in the common central region for both type 1 and type 1b elements, shared only ∼24% amino acid sequence level identity with *virD2* from type 2 TnPZs, where it was located at the left end of the element. No *virD2* homologs were found in *H. pylori* genomes outside of TnPZs.

Also, present in all full size TnPZs was a gene encoding ParA, whose homologs participate variously in partitioning DNA during cell division, positioning the type IV secretion complex on the cell surface, and amplifying VirD2-nicked DNA that will be transferred by conjugation [22], [23]. Finally, the TnPZ *topA* gene encodes a homolog of topoisomerase I that could affect DNA superhelical density [20], [21].

### orfQ

Each full length TnPZ contained a large (≥2800 codon) gene designated *orfQ* (Fig. 1), for which NCBI conserved domain and SMART (http://smart.embl-heidelberg.de/) algorithms identified domains characteristic of DNA methylases and helicases that might affect DNA replication, repair or transcription. The OrfQ protein of *H. cetorum*'s TnPZ exhibited only 46–48% amino acid identity to *H. pylori* OrfQ proteins, contained numerous internal insertions and substitutions, and was >4000 amino acids long. Identities among *H. pylori* OrfQ proteins ranged from 92 to 98%, but no phylogenetic clustering of *orfQ* alleles was found, in contrast to that seen with *xerT*, described above.

In terms of genome sequenced reference strains, *orfQ* was absent from HPAG1 [32] (this strain lacked a plasticity zone); most of it was also missing from strain 26695 [18], although its 3′-end is represented in the 61 codon orf *hp0999*; and it was broken into three adjacent orfs in the published genome sequence of strain J99 (*jhp0927*, *jhp0928* and *jhp0929*) [8]. Recently sequenced strains G27 and P12 had full-length copies of *orfQ*. Homologs with similar domain architecture are found by NCBI - BLASTP searches in a subset of strains of many pathogens, Gram-positive as well as Gram-negative.

### TnPZs can affect bacterial phenotypes

Preliminary experiments had indicated that PeCan18B and HUP-B43 could each colonize FVB/N mice for up to eight weeks, with mean recoveries of ∼1,000–2,000 *H. pylori* colony forming units (cfu)/stomach. To learn if TnPZs were needed in vivo, FVB/N mice were inoculated with deletion derivative strains, in which the entire element had been replaced with a chloramphenicol resistance determinant (*ΔTnPZ*). These *ΔTnPZ* strains were each found to colonize mice at about the same density as their wild type parents, scored either two or eight weeks after inoculation (average of 1,000–2,000 cfu per stomach, five mice with each strain for each time point). This indicated that TnPZs are not essential, in agreement with their absence from the genome sequence of *H. pylori* strain HPAG1 and the genome sequenced strain of *H. acinonychis* [32], [33], and the inability to PCR amplify TnPZ genes in 16 other *H. pylori* strains (8 of 102 from global strain collection; 8 of 44 Shimaa strains) noted above.

**Figure 5:**
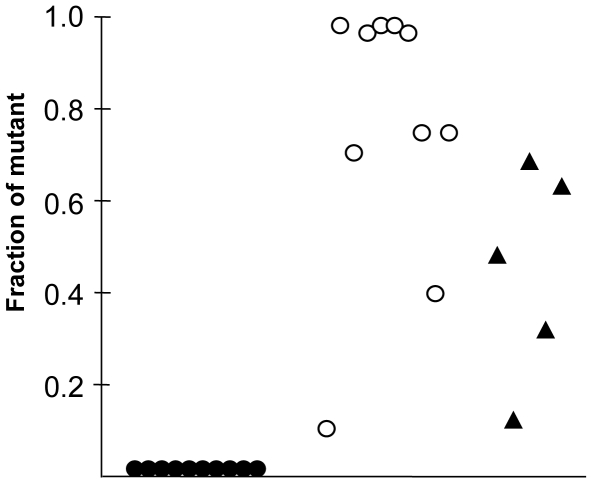
TnPZs can contribute to fitness in mice. Animals were infected with 1∶1 mixtures of mutant and isogenic wild type strains, sacrificed two weeks later, and scored for abundance of each strain type by tests of individual colonies for susceptibility/resistance phenotypes. Black circles represent mixed infection with PeCan18B wild type and PeCan18B *ΔTnPZ(cat)*; white circles represent mixed infection with PeCan18B wild type and PeCan18B *Δtfs3(kan)*; black triangles represent mixed infection with HUP-B43 wild type and HUP-B43 *ΔTnPZ(cat)*. Each dot represents *H. pylori* types (fraction of mutant relative to wild type) recovered from a single mouse.

Competition experiments were used to examine fitness more critically: each *ΔTnPZ* derivative was inoculated along with its isogenic wild type parent in a 1∶1 mixture; the mice were sacrificed two weeks later; *H. pylori* were cultured from separated gastric antrum and corpus tissues; and single colonies were picked and scored for chloramphenicol resistance, to distinguish *ΔTnPZ* mutant and wild type parent strains. No significant effect of the *ΔTnPZ* allele was detected in HUP-B43 (median yield of *ΔTnPZ*, 50–60% of total) (Fig. 5, triangles). In contrast, the *ΔTnPZ* allele sharply reduced PeCan18B's competitive ability: none of the 20 colonies picked per mouse in any of ten mice tested was Cam^R^, nor were any Cam^R^ colonies recovered by direct streaking of infected stomachs on chloramphenicol-containing agar (Fig. 5, filled circles). Equivalent competition tests of a PeCan18B *Δtfs3* derivative and PeCan18B wild type indicated that *Δtfs3* increased fitness in this mouse model: with 12 of 14 such mixedly infected mice at least 80% of recovered *H. pylori* colonies were of the *Δtfs3* type. Only with two of the 14 mice was wild type more abundant than its *Δtfs3* mutant derivative (Fig. 5, open circles).

**Figure 6:**
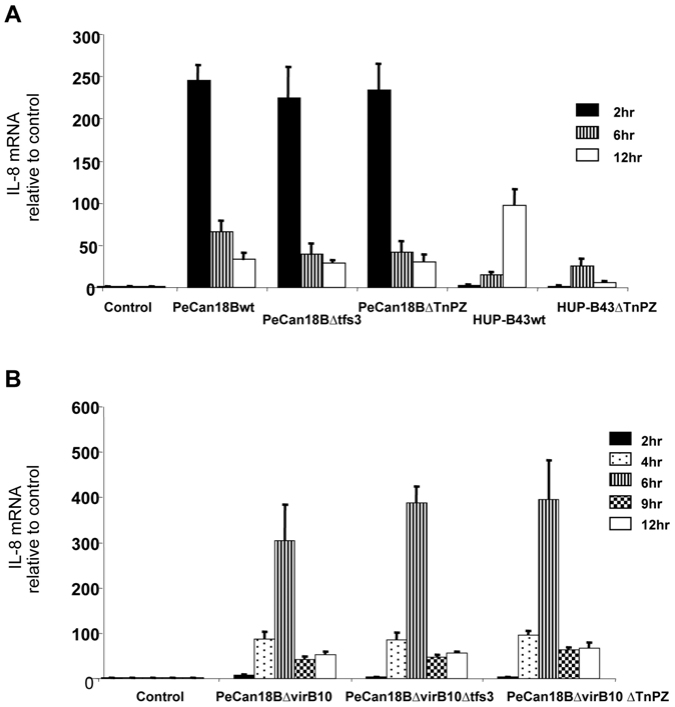
Induction of IL-8 transcription in AGS cells. AGS cells were infected with wild type or isogenic mutant *H. pylori* strains or were mock infected (Control), and levels of IL-8 mRNA, normalized relative to β-2 microglobulin mRNA, at the indicated times were determined by real time PCR with primers listed in Table S1D. These analyses showed that AGS cell infection by strain PeCan18B caused strong rapid IL-8 induction, and that this induction depended on the virB10 gene of this strain's *cag PAI*, but not on *tfs3* nor TnPZ; in contrast, infection by HUP-B43, which naturally lacks a *cag PAI* caused a gradual, TnPZ-dependent IL-8 induction.

In a second experiment, cultured AGS human gastric epithelial cells were inoculated with wild type or isogenic *ΔTnPZ* mutant strains, RNA was extracted, and real time PCR was used to estimate the abundance of the proinflammatory cytokine IL-8 transcript, and thereby assess if either TnPZ affects inflammatory pathways. HUP-B43 lacks the *cag PAI*, a virulence gene cluster implicated in a strong and rapid IL-8 induction [10], [12], [26]. Fig. 6A shows that AGS cell infection by strain HUP-B43 resulted in an approximately 100-fold increase in IL-8 transcripts, but only at ∼12 hours post-infection, not at ∼two hours, as was characteristic of *cag PAI*-mediated IL-8 induction. This delayed induction depended on HUP-B43's TnPZ.

PeCan18B did contain an intact *cag PAI*, unlike HUP-B43, and strongly induced IL-8 transcription by two hours post-infection, but this stimulation was transient (no longer evident by six hours post-infection (Fig. 6A)). This early and rapid induction required an intact *cag PAI* (*virB10*-homolog), but not this strain's TnPZ or its *tfs3* component. However, the *ΔvirB10* allele allowed a later strong increase in IL-8 transcripts, peaking at about six hours post infection. This secondary IL-8 induction was not affected by deletion of this strain's TnPZ (Fig. 6B), and thus may be due to another virulence determinant, such as the *oipA* or homB genes [45], [46].

In conclusion these experiments indicate that the TnPZs can affect bacterial phenotypes in at least two ways, depending on TnPZ gene content, and perhaps background bacterial genotype as well, since HUP-B43 and PeCan18B are not related to one another.

## Discussion

We found that *H. pylori's* plasticity zones, the chromosomal DNA segments initially postulated to be regions of high genetic variability [8], [9], consist of novel transposons (TnPZs) or remnants of them, and that they are abundant in *H. pylori* populations worldwide. The full-length apparently active transposons in many bacterial lineages are more stable than had been implied by the term “plasticity zone”, which was devised long ago, when only two H. pylori genome sequences were available [8]. We found TnPZs at numerous chromosomal sites, in each case flanked by direct repeats of 5′AAGAATG or a closely related sequence, one copy of which was generally present at unoccupied sites in other strains. Several TnPZ types (1, 1b, 2) were distinguished based on differences in DNA sequence and gene order. Each encoded many of the same functions and each was inserted at targets containing 5′AAGAATG or a closely related sequence. The TnPZ terminal sequences, which probably contain sites of transposase action, differed markedly among element types, but in each case, contained imperfect inverted repeats (Fig 2). The constancy of this feature suggests that it is important functionally.

### Proposed transposition mechanism

We hypothesize that TnPZs are conjugative transposons whose movement between cells occurs via TnPZ-encoded type IV protein secretion complexes. Conjugation is used by numerous transposable elements to spread in bacterial populations. Many of these “integrative conjugative elements” (or “ICE”) [3], [41] insert preferentially at just one site per bacterial genome. This specificity is epitomized by the 100 kb *V. cholerae* SXT element, whose insertion is mediated by a phage lambda integrase-like protein, a member of one major subfamily of tyrosine recombinases [3], [41], [47]. TnPZs, in contrast, insert into many sites, at the limit perhaps any site containing a 5′-AAGAATG target sequence. We propose that TnPZ insertion is mediated by their encoded XerT proteins, which belong to a subfamily of tyrosine recombinases, distinct from those containing lambda phage and SXT integrases. Insertion with TnPZ's heptanucleotide specificity would be a novel action for Xer-related proteins, however, since the prototype (*E. coli*) XerC and XerD proteins are highly specific for a longer unique chromosomal sequence, the ∼28 bp *dif* site. The *E. coli* XerC and XerD proteins convert dimeric chromosomes to monomers before cell division by acting in a complex with the cell septation protein FtsK on *dif* site DNA: XerD forms Holliday junctions using one strand of the interacting *dif* sites if it is activated by FtsK protein; and XerC then forms these junctions using the other strand [48]. In a variant process, used by the single stranded *Vibrio cholerae* phage CTXphi, XerC complexed with catalytically inactive XerD forms Holliday junctions between one DNA strand in a hairpin (inverted repeat) structure in phage DNA and one strand at the chromosomal *dif* site. These latter junctions are resolved by replication to generate a fully integrated CTXphi prophage [49]. Future experiments will test (i) if TnPZ is a conjugative element, (ii) what form of TnPZ DNA (e.g., single strand, hairpin or fully double stranded) is used as a substrate for insertion, (iii) if TnPZ XerT is really the transposase, and if so, (iv) what interaction partners, if any, are also involved in transposition, and (v) how its unusual heptanucleotide specificity is achieved.

### TnPZ adaptive traits

Transposable elements are well known to increase in populations by transposition per se, a property termed “selfish DNA” [50]; their abundance is further enhanced if they also contribute to bacterial fitness [51]. We began to examine these issues by generating deletions of TnPZs and of an embedded *tfs3* gene cluster. One strain was found whose TnPZ contributed to the vigor of bacterial colonization in a short-term mouse infection model, whereas its *tfs3* cluster seemed to diminish fitness (PeCan18B); and another strain was found whose TnPZ stimulated transcription of the IL-8 cytokine genes in cell culture but did not affect fitness in equivalent mouse infections (HUP-B43). The genes and factors responsible for these effects are not known, but our interest, for future studies, is especially drawn to the immense orfQ gene (>2800 codons in *H. pylori*; >4,000 codons in *H. cetorum*), whose encoded protein's helicase and DNA methylase domains suggest possible effects on gene expression. BLASTP searches identified OrfQ-related proteins with similar domain structures encoded in the genomes of diverse Gram-positive and Gram-negative pathogens. Analyses of OrfQ protein synthesis, stability and localization (in bacterial and/or host cells), and effects of OrfQ and its component domains on DNA methylation, gene expression patterns or chromosome (chromatin) dynamics may give valuable insights into bacterial growth and/or interaction with host tissues or disease. Equivalent studies of other selected TnPZ-encoded proteins are also warranted.

### TnPZ transmission and stability

The occurrence of substantially the same type 1 TnPZ at multiple genomic sites in Shimaa village strains, and of a similar element in the unrelated Italian strain G27 suggested that these elements are active and can be stable, potentially for the many thousands of years since *H. pylori*-infected humans migrated from Africa to the Eurasian continent, and eventually to The Americas [13], [52], [53]. Alternatively these TnPZs might have spread rapidly after a recent chance introduction, e.g., of a European TnPZ into the Shimaa population. We do not favor this latter alternative because the village of Shimaa is remote and rarely visited by outsiders; and sequences of Shimaa strain housekeeping and virulence genes belong to a distinct Asian-like phylogenetic cluster, with no admixture detected of European allele types that are abundant in urban Peruvian strains (Kersulyte et al., manuscript in preparation). Recent movement of an Amazonian transposon into the Italian *H. pylori* population also seems unlikely. Similar considerations apply to the type 2 TnPZs in Shimaa, urban Peruvian and German *H. pylori* strains, and also in an isolate of *H. cetorum*. Because the Beluga whale that was the source of the sequenced *H. cetorum* TnPZ was from an aquarium, one could suspect recent TnPZ transmission from a human *H. pylori* strain. However, we had also found a *jhp0940*-like sequence in another *H. cetorum* strain that had been cultured from a wild dolphin. It was 97% identical to the *jhp0940-like* orf *pz44w* found in the whale *H. cetorum* strain, and also 91% identical to corresponding sequences in *H. pylori* strain J99 in the 775 bp sequenced (unpublished data). This indicates that TnPZs may also be ancient members of the *H. cetorum* gene pool. Further studies of H. cetorum (marine mammal) isolates and related species are warranted.

The multiple remnant and internally deleted TnPZ elements found in 26695, J99 and several other strains, and our PCR-based population data indicate that segments of TnPZs can be lost by both spontaneous and transposable element mediated deletion processes, much as has been seen with normal chromosomal genes [54]. It is attractive to imagine that chance inactivation of the ability to transpose facilitates the accumulation of deletions and other mutations. TnPZs might also be unstable in some *H. pylori* strains due to more active rearrangement-generating transposable elements [16], [17], or to lower fidelity of DNA replication or repair and thus higher rates of spontaneous mutation [55]. The basis for finding well conserved, apparently stable TnPZ elements in some strains and only remnants in others, and mechanisms underlying these differences merit closer examination.

In conclusion, we have discovered a novel class of *Helicobacter* transposons; identified their remnants in complex mosaic structures in certain strains; and found that these elements can affect bacterial phenotypes in at least two ways (fitness in mice; induction of proinflammatory IL-8 synthesis) depending on TnPZ gene content and perhaps bacterial gene content as well. These results open up new questions of fundamental interest. Their further study should enrich our understanding of infection and disease processes, the diversity of transposition mechanisms and regulation, and of genome evolution.

## Materials and Methods

### Ethics Statement

Some of the *H. pylori* strains studied here were cultured from gastric biopsy specimens from residents of the village of Shimaa in the remote Peruvian Amazon who were symptomatic and had accepted an offer of diagnostic endoscopy. Endoscopy was preceded by explanation and discussion of the procedure, risk and anticipated uses of the biopsies – first with the village chief, and then with villagers. These explanations and discussions were carried out in Spanish and also in native Machiguenga languages, with the aid of a Spanish-Machiguenga interpreter, and in the presence of a trusted physician from the Peruvian Ministry of Health (in residence in the village for two years). The endoscopies were performed with informed consent (written or verbal, depending on participant's literacy) for bacterial culture and genetic analyses as described here under protocols approved by the Human Studies Committees of Johns Hopkins University, of AB Prisma, and of Universidad Peruana Cayetano Heredia. These three institutional review board committees had, in particular, approved both the endoscopy procedure and bacterial culture and genetic analysis experiments.

Mice of the wild type inbred FVB/NJ line were maintained in the Washington University Medical School Animal Quarters (Animal Welfare Assurance #A-3381-01) with water and standard mouse chow ad libidum, and used in protocols of bacterial inoculation by oral gavage and mouse sacrifice two weeks later that had been approved by the local Animal Studies Committee (approval #20040004).

### General methods

*H. pylori* was grown on brain heart infusion agar (Difco) containing 10% horse blood in a microaerobic (5% O2, 10% CO2) atmosphere following standard protocols [16], [56]. Chromosomal DNA for routine PCR and sequence analysis was isolated using the QIAamp DNA Mini kit (Qiagen, Chatsworth, CA). Genomic DNA of higher molecular weight, which is needed for efficient direct chromosomal sequencing [16], [24], was isolated by a hexadecyltrimethylammonium method [57]. PCR amplification, product purification, and DNA sequencing, both of PCR products, and directly from chromosomal DNA, were carried out as described [16], [24]. DNA sequence editing and analysis were performed with programs in Vector NTI (Informax, Bethesda, MD); programs and data in *H. pylori* genome sequence databases, and Blast homology search programs (http://www.ncbi.nlm.nih.gov/blast/blast.cgi). Unrooted trees were constructed by Neighbor-Joining (Mega 3.1, http://www.megasoftware.net/).

### Bacterial strains

The 102 *H. pylori* strains used in a population survey of the distribution of plasticity zone genes were from the Berg lab collection [24], [58]. The strains used for plasticity zone sequencing were as follows: PeCan18B (*cagPAI*+, *tfs3*+) from an urban Peruvian gastric cancer patient; HUP-B43 (lacking *cagPAI* and *tfs3*) from a Spanish gastritis patient, kindly provided by Drs. M. Lopez Brea and T. Alarcon; Shi470 and Shi170, from symptomatic residents of the ∼600 inhabitant Machiguenga (Amerindian) village of Shimaa in the remote Peruvian Amazon; strains MIT-00-7128 and MIT-99-5656 of the distinct species *Helicobacter cetorum*, cultured from a captive beluga whale (Mystic Aquarium, Conn) and a beached wild dolphin, respectively, kindly provided by Dr. James Fox [59]. PeCan18B and HUP-B43 were chosen after each had been found to colonize FVB/NJ mice (unpublished data). Shi470 and Shi170 were members of a 44-strain collection of independent clinical isolates cultured from gastric biopsy specimens from symptomatic Shimaa village residents who had accepted an offer of diagnostic endoscopy.

### TnPZ sequence analysis

Four TnPZs were sequenced using PCR and chromosomal walking strategies [16], [24], and one was identified by inspection of our recently determined genome sequence of strain Shi470 (GenBank Accession CP001072). The PeCan18B and HUP-B43 TnPZs were chosen as representative of elements containing and lacking a *tfs3* type IV secretion gene cluster (Figs. S3 and S8, respectively), and because the strains carrying them had been found to colonize FVB/N mice. A third TnPZ was from a strain of the separate species *H. cetorum* from a Beluga whale [59], and was chosen after an unexpected success in PCR amplification with primers specific for *H. pylori* plasticity zone genes *jhp0931, jhp0940* and *jhp0941* (Fig. S7). A fourth TnPZ was identified in our genome sequence of Shi470, one of 44 strains Shimaa village strains, noted above (Fig. S1). The fifth TnPZ was from a different Shimaa village strain (Shi170), which contained two distinct TnPZs, one closely related to that in Shi470 by PCR criteria, and the second distinct TnPZ (Fig. S4). TnPZs found in two other genomes whose sequences were released during preparation of this manuscript (G27, GenBank Accession CP001173; and P12, which has two TnPZs, GenBank Accession CP001217) were also analyzed (Figs. S2, S5 and S6). Database searches and Blast analyses used in this report were completed in April and May 2009.

### Construction of TnPZ and *tfs3* deletion derivatives

Deletions of the TnPZs of PeCan18B and HUP-B43 and of PeCan18B's *tfs3* gene cluster were done by PCR without cloning to replace these segments with kanamycin and chloramphenicol resistance determinants, and then used for transformation [16], [24] (primers in Table S1C). It is important for the experiments presented below, that the recipient populations had, in each case, been recently recovered from infected FVB/N mice; and that *ΔTnPZ* and *Δtfs3* transformants used to inoculate mice were then collected as pools, not single colonies. These precautions reduce risks of attenuation of mouse colonizing ability by any chance unfortunate choice of particular single colonies or passage in culture. In addition, the deletion mutants used later in competition tests were recovered as pools from infected mice, to further reduce risks of attenuation.

### Mouse infection

Young (8–10 week) FVB/NJ mice were purchased from Jackson Laboratories (Cat. No. 001800). Young overnight *H. pylori* cultures were suspended in phosphate-buffered saline (PBS) at densities of approximately 2×10^9^ colony forming units (cfu) per ml, and 0.5 ml of suspension was used for inoculation, as described [56]. In cases of mice inoculated with two strains, the 0.5 ml suspension contained equal amounts of each strain (final concentration, 2×10^9^ cfu per ml). Colonization was generally scored two weeks after inoculation. *H. pylori* quantitative culture entailed homogenizing the tissues in 0.2 ml of PBS using a disposable Pellet Pestle (Kontes), and spreading serially 10-fold diluted aliquots of each suspension on BHI agar selective medium containing six antibiotics (amphotericin B, trimethoprim, vancomycin, nalidixic acid, polymyxin B and bacitracin) [56] to inhibit growth of microbes other than *H. pylori*, and incubated as above. In cases of mice jointly infected with deletion mutant and wild type *H. pylori* strains, the relative abundance of each strain was estimated by testing ≥20 single colonies per mouse for resistance to chloramphenicol or kanamycin, as appropriate.

### IL-8 induction in AGS cell line

Cells of the AGS human gastric epithelial line (American Type Culture Collection, Manassas, VA) were grown in RPMI1640 medium with L-glutamine (Cellgro, Herndon, VA) containing 10% heat-inactivated fetal bovine serum (Sigma, St. Louis, MO) in six well dishes for 24 hr (2 ml per well). Cells were then washed three times with antibiotic-free medium and infected with 24 hr old *H. pylori* cultures (ratio 1 AGS cell/250 bacterial cells) for times appropriate to the experiment. Total RNA was isolated from uninfected (control) or infected AGS cells using Trizol reagent, and reverse transcribed with Superscript II Reverse Transcriptase with random hexanucleotide primers (Invitrogen, Carlsbad, CA). The resultant single–strand cDNAs were then used for Real Time PCR with Jumpstart Taq DNA polymerase (Sigma, St. Louis, MO) and primers listed in Table S1D. Crossing threshold values for cytokine IL-8 mRNA were normalized to beta-2-microglobulin. Changes in mRNA levels were expressed as fold change, relative to control (beta 2-microglobin).

### DNA sequence GenBank Accessions

A 56,651 nt sequence that contained strain PeCan18B's plasticity zone (TnPZ) and flanking DNA was deposited as GenBank Accession AF487344 (plasticity zone coordinates 864–53286: type 1b remnant, 864–3917; and type 2 element, 3918–53286). A 33,671 nt sequence that contained strain HUP-B43's TnPZ and flanking DNA was deposited as GenBank Accession AY487825 (TnPZ coordinates 1285–28713). A 64,162 nt sequence that contained *Helicobacter cetorum* strain MIT-00-7128's TnPZ and flanking DNA was deposited as GenBank Accession EU015081 (TnPZ coordinates 3698–58762). The full genome sequence of strain Shi470 from an Amerindian (Machiguenga) resident of Shimaa village (Peruvian Amazon), was deposited as GenBank Accession CP001072 (TnPZ coordinates 874704–913876). A 47,447 nt sequence that contained Shimaa village strain Shi170's TnPZ was deposited as GenBank Accession EU807988 (TnPZ coordinates 574–47093). Sequences between the 5S,23S rRNA - *ftsZ* (*jhp0913/hp0979*) gene (location occupied by TnPZs or remnants in 26695, J99, PeCan18B and HUP-B43) in *H. pylori* strains HUP-B41 (Spain), HUP-B62 (Spain), I-49 (India), Santal-52 (India), PeCan14B (Peru), CPY6021 (Japan) and PeCan16A (Peru), which in each case corresponds to an empty site, were deposited as Genbank Accessions EU019081- EU0190817, respectively.

## Supporting Information

Table S1 Oligonucleotide primers.
(0.16 MB DOC)

Table S2 **Results of PCR based survey of plasticity zone genes in 102 *H. pylori* strains and *H. cetorum*.** Sequences of PCR primers used are listed in Table S1A. The following sets of strains were tested: 22 from Japan (11 gastric cancer, 11 gastritis), 24 from urban Peru (Lima region shantytown) (12 from gastric cancer, 12 from gastritis), 24 from Spain (12 *cagPAI* ^+^, 12 *cagPAI* ^−^), 22 from India, 10 from Gambia and one *H. cetorum* (from Beluga whale). DNAs from these strains were tested with pairs of primers specific for genes in plasticity zones of reference strains 26695 and J99, and also Peruvian PeCan18B (*pz32*) and Japanese CPY6081 (*jhp926*≈, called *jhp926like* in GenBank Accession AY128680). Subsequent discoveries that these genes are from one of several types of transposons (TnPZs) (see text) allowed the indicated classifications: type 1 (3 pairs of primers), type 1b (7 pairs), mix for type 1/1b (1 pair), mix for type 1/type 1b/type 2 (1 pair) and type 2 (4 pairs) TnPZs. The analysis of the *tfs3* component from type 2 TnPZs with 14 pairs of primers was reported previously [1]. In overview, 32% of the 102 strains yielded PCR products indicative of all three TnPZ types, 32% of two types, 27% of one type; and 8% did not yield PCR products with any primer pairs, and thus may have been TnPZ-free. The PCR data indicated that many strains contained TnPZ fragments, not complete TnPZs. Three orfs (*jhp0940*, *jhp0947* and *jhp0949*), although associated with gastric pathology in some studies [2]–[5] were not significantly associated with gastric cancer in our study: *jhp0940* was present in four of 12 Peruvian gastritis strains and two of 12 Peruvian gastric cancer strains; and although absent from 11 Japanese gastritis strains, was present in only one of 11 Japanese gastric cancer strains. Similarly, *jhp0947*, which was correlated with gastric cancer in other studies, was equally present in Peruvian gastric cancer and gastritis strains (present in four of 12 strains of each group), was absent from Japanese strains. *jhp0949* was present in four of 11 Japanese gastric cancer and five of 11 gastritis strains and in five of 12 Peruvian gastric cancer as well as gastritis strains.
(0.34 MB DOC)

Figure S1 Type 1 TnPZ in Peruvian Amazon Shimaa village strain Shi470. This TnPZ is 39 kb long, has 33 orfs and is inserted into the homolog of strain 26695 gene *hp0488* (TnPZ coordinates 874704–913876 bp, orfs *hpsh_04480 - hpsh_04640* in this strain's full genome sequence; GenBank Accession CP001072). It has a novel 5.8 kb segment, not represented in strains 26695 or J99 (*hpsh_4550* through 3′ half of *hpsh_4590*), but which is present in other type 1 TnPZs and in the type 1b TnPZ of German strain P12. The Shi470 type 1 TnPZ contains a type IV protein secretion system gene cluster (*hpsh_04545 - hpsh_4640*) that is similar in size (16.3 kb), gene content and arrangement to *tfs3* in type 2 TnPZs [1], but with very low, if any, DNA sequence identity to them. It was therefore designated *tfs3a*. Genes in *tfs3a* that resemble those in canonical type IV secretion systems are: *virD4 (hpsh_04545), virB11 (hpsh_04565), virB9 (hpsh_04595), virB8 (hpsh_04600), virB7 (hpsh_04605), virB4 (hpsh_04615), virB3 (hpsh_04620), virB2 (hpsh_04625)*. Symbols. Boxes above the line, orfs transcribed rightward; below the line, transcribed leftward. Box color coding: green, TnPZ orfs with homologies to reference strain 26695 (orf numbers start with *hp*); red, TnPZ orfs with homologies to reference strain J99 (orf numbers start with *jhp*). Orfs marked with ≈ have relatively low protein level identity (35–70%) to their homologs in reference strains 26695 and J99; protein level identities of other orfs to those of one or the other reference strains range from 90–95%. Boxes in light blue - Shi470 strain specific orfs, not found in strains 26695 or J99. Above the line, homologies to reference strains; numbers below the line, orf designations as in GenBank annotation (preceded by *hpsh_*).
(1.16 MB TIF)

Figure S2 Type 1 TnPZ in the genome sequence of Italian strain G27. This 39 kb element (coordinates 1045701–1085075 bp in strain G27 genome sequence of Baltrus et al.; GenBank Accession CP001173) is very similar to the type 1 TnPZ in strain Shi470 (95% DNA identity across its length without gaps). Numbers below the line (986 through 959), orf designations in strain G27 GenBank annotation (preceded by HPG27_). The DNA sequences of some of the TnPZ orfs annotated in Shi470 but not annotated in G27, are nevertheless present (*hpsh_04605, hpsh_04585, hpsh_04575, hpsh_04540*). if a gene is predicted to be inactive (due to a stop codon or frameshift), it is marked with an asterisk (*). For other symbols, see legend to Fig. S1.
(1.10 MB TIF)

Figure S3 Type 2 TnPZ and type 1b TnPZ remnant in urban Peruvian strain PeCan18B. The segment containing TnPZ sequences in PeCan18B is 52.4 kb long (coordinates 864–53286 bp in GenBank Accession AF487344; genes also designated *pz1* through *pz41;pz* gene numbers below line), and includes two segments of novel DNA, not known from reference strains 26695 or J99 (in dots): 10.8 kb in its *tfs3* component (3′-end of *orfH* through *orfP*) and 4.7 kb of additional sequence (*pz30* through 5′-end of *pz33*). These sequences are also present in other full size type 2 TnPZs. The type 2 TnPZ in PeCan18B extends from the genes designated *pz5 - pz41*, and is inserted next to a remnant of a type 1b TnPZ (genes *pz1 - pz4*, homologs of *jhp0951 - jhp0949*). This structure is inferred to have resulted from type 2 TnPZ insertion into a resident type 1b element, and deletion adjacent to one type 2 TnPZ end. Sites of IS*Hp608* element insertion and their orientations of are indicated. Boxes in dots, orfs originally found in PeCan18B (*tfs3* orfs H - P, *pz30-pz33*), but present in all full size type 2 TnPZs. TnPZ orfs with homologies genes found in both 26695 and J99 are indicated in black. For other symbols, see legends to Figs. S1 and S2.
(1.14 MB TIF)

Figure S4 Type 2 TnPZ in Peruvian Amazon Shimaa village strain Shi170. This 46.5 kb long TnPZ has 34 orfs, 16 of which belong to *tfs3* (TnPZ coordinates 574–47093 bp in GenBank Accession EU807988). It is similar to the type 2 TnPZ in PeCan18B in gene content and arrangement, except that the *hp0993* and *hp0944* homologs in PeCan18B were replaced by homologs of *hp0712* and *hp0713*, which are not within the plasticity zones of reference strains J99 or 26695. This TnPZ is inserted between homologs of J99 genes *jhp0210* and *jhp0211*. Shi170 also contains a type 1 TnPZ that is closely related by PCR criteria to the sequenced element in Shi470. For symbols, see legends to Figs. S1 - [Supplementary-material pone.0006859.s004] S3.
(1.12 MB TIF)

Figure S5 Type 2 TnPZ in German strain P12. This TnPZ is just 30 kb long (coordinates 1394773–1424783 bp in strain P12 genome sequence of Fischer et al. GenBank Accession CP001217), has 32 annotated orfs (*HPP12_1320 - HPP12_1353*) and contains three deletions relative to other type 2 TnPZ elements such as that in PeCan18B (absence of *pz21 - pz23, pz29 - pz31 and pz33 - pz34*, Fig. S3). This type 2 TnPZ is inserted into a homolog of jhp1272 (putative) and co-exists in this strain with a full length type 1b TnPZ. Sites of deletion are marked with open triangles; for other symbols, see legends to Figs. S1 - [Supplementary-material pone.0006859.s004] S3.
(1.04 MB TIF)

Figure S6 Type 1b TnPZ in German strain P12. This TnPZ is 40.8 kb long (coordinates 452020–492773 bp in GenBank Accession CP001217 of Fischer et al), has 35 orfs (*HPP12_0437 - HPP12_0473*) and is inserted into a homolog of *hp0464* (hsdR) of strain 26695. This type 1b TnPZ is similar in gene organization to type 1 TnPZs found in strains Shi470 (Fig. S1) and G27 (Fig. S2), although it differs markedly from them in sequence for ∼8 kb from each end. Included among the divergent genes are *xerCD* on the left, and the *virB8 - virB2* segment of the type IV secretion gene cluster, here called *tfs3b*. The topA and *virB4* genes contain inactivating mutations and thus are pseudogenes. For symbols, see legends to Figs. S1-[Supplementary-material pone.0006859.s004] S3 and S5.
(1.13 MB TIF)

Figure S7 Type 2 TnPZ in H. cetorum strain MIT-00-7128. This TnPZ is 55 kb long (coordinates 3698–58762 bp in GenBank Accession EU015081) and has 46 orfs, 16 of them belong to *tfs3*. It is inserted in a strain-specific region, but close to homologs of restriction-modification genes on one side and a GTP-binding protein gene on the other (*hp0303/jhp0288*). Although this *H. cetorum* strain was cultured from a Beluga whale, its TnPZ had a striking resemblance to the type 2 TnPZs of *H. pylori* (PeCan18B, Shi170) based on sequence homology and gene arrangement. Nevertheless, it is also unique in containing 8.5 kb sequences not found in other known TnPZs (in white). 3.5 kb of unique DNA is distributed among three parts of the huge *orfQ* (putative helicase/methyltransferase) gene (4043 codons, *pz17w*, vs. 2879 codons, *pz21*, in PeCan18B). The other 5 kb is scattered among several clusters of short orfs (*pz18w - pz21w, pz22w - pz25w, pz32w -pz34w, pz43w*). For symbols, see legends to Figs. S1-[Supplementary-material pone.0006859.s004] S3 and S5.
(1.11 MB TIF)

Figure S8 Type 1b TnPZ in Spanish strain HUP-B43. This TnPZ is just 27.4 kb long (coordinates 1285–28713 bp in GenBank Accession AY487825), and has 21 orfs, two IS*Hp608s* and one IS*Hp609*. It has a complete *virD4* homolog (*pz13b*) whose 3′-end closely resembles a truncated version in the strain 26695 genome (*hp1006*). This TnPZ has a large internal inversion (*pz4b - pz13b*) relative to the type 1b TnPZ of strain P12 (orfs *HPP12_0439 - HPP12_0444* on the left; orfs *HPP12_0455 - HPP12_0470* on the right) with multigene deletions at each inversion breakpoint. One inversion/deletion breakpoint coincides with an IS*Hp608* insertion (*pz14b*, truncated version of *jhp0950*). No IS element is precisely at the other breakpoint, although one might have been present earlier and then become lost by subsequent deletion. The insertion of IS*Hp608* in *pz3b* is not associated with any deletion. For symbols, see legends to Figs. S1-[Supplementary-material pone.0006859.s004] S3 and S5.
(0.91 MB TIF)

Figure S9 Region between ftsZ gene and the 5S-23S rRNA locus. Color coding as in Figs. S1-[Supplementary-material pone.0006859.s004] [Supplementary-material pone.0006859.s005] [Supplementary-material pone.0006859.s006] [Supplementary-material pone.0006859.s007] [Supplementary-material pone.0006859.s008] [Supplementary-material pone.0006859.s009] S8; other colors represent strain specific sequences, sometimes shared by other strains but absent in the 26695 or J99 genome sequences. This region has been used frequently for TnPZ insertion. A. Region between *ftsZ* and 5S-23S rRNA without TnPZ insertion (“empty site”), where TnPZ is inserted in other genomic locations or is absent (HPAG1, PeCan16A). Seven strains were PCR amplified with primers located in ftsZ and 5SrRNA (979+5S, PCR size 1.6 to 4.6 kb, Table S1A) and sequenced (GenBank Accession EU019081 - EU019087), five of which are presented here (Japanese CPY6021, 2.3 kb; Spanish HUP-B41 and B62, 3.6 kb; urban Peruvian PeCan16A, 3.6 kb and Indian I-49, 4.2 kb). Other sequences were taken from genome annotations (HPAG1, 4 kb; P12, 14 kb; G27, 13 kb and Shi470, 7 kb). Shi470 has a remnant of IS606 (*orfA* absent, *orfB* with deletion) next to *ftsZ*. Orf numbers below in HPAG1, P12, G27 and Shi470 refer to those used in genome annotations. B. Region between ftsZ and 5S,23S rRNA with sites of insertion of TnPZ remnants marked by arrows in strains 26695 (in total 20 kb), J99 (3.6 kb), HUP-B43 (4.5 kb) and PeCan18B (4 kb).
(1.78 MB TIF)

Figure S10 Structural predictions and comparisons for XerCD-like proteins. This multiple sequence alignment was created for XerT proteins encoded by *H. pylori* TnPZ types 1, 2 and 1b (from strains Shi470, PeCan18B and P12, respectively), chromosomal XerH from Shi470 strain (HPSH_03490) and the XerD and XerC proteins of *E. coli*, using ClustalW. The alignment was validated and slightly refined based on comparing secondary structures predicted by PSI-PRED algorithms (http://bioinf.cs.ucl.ac.uk/psipred/). The observed *E. coli* XerD secondary structure (purple, predicted alpha helix; underline, observed alpha helix; green, predicted beta sheet; bold, observed beta sheet) is also depicted on the figure [6]. Note that regardless of the lack of sequence similarity in the N-terminal domain, the secondary structures align well throughout the sequences. This suggests that all four *H. pylori* proteins have folds similar to that of *E. coli* XerD. Boxed sequence indicates residues implicated in DNA binding for the *E. coli* XerD protein; arrowhead indicates the catalytic tyrosine residue.
(4.52 MB TIF)
