# Supplementary material for: Helicobacter Pylori's Plasticity Zones Are Novel Transposable Elements
Source: PLoS One. 2009 Sep 3;4(9):e6859. doi: 10.1371/journal.pone.0006859 (PMC2731543; doi:10.1371/journal.pone.0006859)
Supplement: Table S1 — Oligonucleotide primers. (0.16 MB DOC) [file pone.0006859.s001.doc]

**Table S1. Oligonucleotide primers.**

**Table S1A. Primers used for survey of plasticity zone genes in global *H. pylori* strain collection**

| **Primer name** | **Sequence** | **Source** |
| --- | --- | --- |
|  |  |  |
| jhp919F | ctaggtaggattgtaagcgatc | *jhp0919*, J99 |
| jhp919R | cgtgttcttggatttgcttaactc |  |
|  |  |  |
| jhp924F | acttcttttactcttagagcataag | *jhp0924*, J99 |
| jhp924R | ttgcactagcgcattaaaatcatc |  |
|  |  |  |
| jhp926F | caggtttgaacaccttttga | *jhp0926*, J99 |
| jhp926R | cttaagcaagaaccaccaca |  |
|  |  |  |
| ES-3F | gctattttgagctatgctttgt | *jhp0926≈*(*jhp926like*), AY128679 |
| ES-1R | tgaaccaaaatacagagcaag |  |
|  |  |  |
| 13out | tggttttattttaggttggacta | junction *jhp0927-8*, *orfQ*, J99 |
| 13in | ctgcctgttggtcgctagatc |  |
|  |  |  |
| jhp931F | actctgttgtagagactaaaccta | *jhp0931*, J99 |
| jhp931R | gtcatttagacttaacacatcatca |  |
|  |  |  |
| jhp940F | gaaatgtcctataccaatgg | *jhp0940*, J99 |
| jhp940R | cctaagtagtgcatcaagg |  |
|  |  |  |
| jhp945F | tgcaataggcagtggtattgatac | *jhp0945*, J99 |
| jhp945R | agtagtattgcgtttgcttgatg |  |
|  |  |  |
| jhp947F | caagatagagcgtttagtca | *jhp0947*, J99 |
| jhp947R | tgcatagctttgcatttgca |  |
|  |  |  |
| jhp949F | caagattctttagttgtgca | *jhp0949*, J99 |
| jhp949R | cagcatttagcaagttagcag |  |
|  |  |  |
| jhp951F | atgagtgattgtaaaatgagtag | *jhp0951*, J99 |
| jhp951R | gtctaaataatcaaagaattgtc |  |
|  |  |  |
| hp441F | cattgctatcgttgtattgac | *hp0441*, 26695 |
| hp441R | gtattgtaagcacacaagag |  |
|  |  |  |
| hp446F | gaatacaccgtgcttgtttg | *hp0446*, 26695 |
| hp446R | actcatggaacttttaagtg |  |
|  |  |  |
| hp1000F | aaaggagggagcggtaaatc | *hp1000*, 26695 |
| hp1000R | tttgatccttaaatacccgttc |  |
|  |  |  |
| X-F | tgctagaagtcattgagagcatg | *pz32* in PeCan18B, AF487344 |
| X-R | catctcttatgatttgtaagctc |  |
|  |  |  |
| Rbs5S | gaagtcaagctcttcatcgctg | Empty site (between 23S-5S rRNA |
| hp979 | tgatcatgtg cgcgttacta tcat | and *hp0979/jhp0913* genes) |

**Table S1B. Primers used for analysis of Shimaa village strains**

| **Primer name** | **Sequence** | **Source** |
| --- | --- | --- |
|  |  |  |
| **Primers for individual gene segments** | |  |
|  |  |  |
| ShiPZ 824-F | actatagaactagaagcagttatg | *HPSH_04480* |
| ShiPZ 1252-R | caatatccctactcttagcatagac |  |
|  |  |  |
| ShiPZ 7573-F | taatcgtgcaatagtcattgatag | *HPSH_04505* |
| ShiPZ 8171-R | gaagcttaccaatgaagaaccac |  |
|  |  |  |
| ShiPZ 11098-F | agccattgatggaaacgctatttc | *HPSH_04510* (*orfQ*) |
| ShiPZ 11563-R | tgaataagctcttctgaattcatc |  |
|  |  |  |
| ShiPZ 23332-F | acttgatagcttgtaaagacttgca | *HPSH_04545* |
| ShiPZ 23875-R | tgggtaatactatttttgctgaag |  |
|  |  |  |
| ShiPZ 32255-F | tctgtaatattgtgtttctcgcac | *HPSH_04610* |
| ShiPZ 32851-R | hhttcaaactactgctctttccat |  |
|  |  |  |
| ShiPZ 38427-F | ttgataaggacatttgttactagg | *HPSH_04640* |
| ShiPZ 38913-R | gctttacagagagctttgtgagct |  |
|  |  |  |
|  |  |  |
| **PCR walking across TnPZ in Shi470** | |  |
|  |  |  |
| 61-F1 | actcatcgctagccatcaaagac | Left flank TnPZ in Shi470 |
| ShiPZ 1252-R | caatatccctactcttagcatagac |  |
|  |  |  |
| ShiPZ 824-F | actatagaactagaagcagttatg |  |
| PZ470-4485R | tgccaaaaaccttatgaagtcaatc |  |
|  |  |  |
| PZ470-4461F | gattgacttcataaggtttttggca |  |
| ShiPZ 8171-R | gaagcttaccaatgaagaaccac |  |
|  |  |  |
| ShiPZ 7573-F | taatcgtgcaatagtcattgatag |  |
| ShiPZ 11563-R | tgaataagctcttctgaattcatc |  |
|  |  |  |
| ShiPZ 11098-F | agccattgatggaaacgctatttc |  |
| PZ470-15348R | agttgtgtgcctttagtggcattag |  |
|  |  |  |
| PZ470-15324F | ctaatgccactaaaggcacacaact |  |
| PZ470-19528R | caaggctttgttatcgtctctcaa |  |
|  |  |  |
| PZ470-19505F | ttgagagacgataacaaagccttg |  |
| ShiPZ 23875-R | tgggtaatactatttttgctgaag |  |
|  |  |  |
| ShiPZ 23332-F | acttgatagcttgtaaagacttgca |  |
| PZ470-28025R | ggagaataccgcttaggaattgct |  |
|  |  |  |
| PZ470-28002F | agcaattcctaagcggtattctcc |  |
| ShiPZ 32851-R | hhttcaaactactgctctttccat |  |
|  |  |  |
| ShiPZ 32255-F | tctgtaatattgtgtttctcgcac |  |
| PZ470-35546R | caaagatggcgtgagtaaatacgcta |  |
|  |  |  |
| PZ470-35521F | tagcgtatttactcacgccatctttg |  |
| ShiPZ 38913-R | gctttacagagagctttgtgagct |  |
|  |  |  |
| ShiPZ 38427-F | ttgataaggacatttgttactagg |  |
| 61-R2 | gaaagcctaaaagatttgagcgag | Right flank TnPZ in Shi470 |
|  |  |  |
| **Flanking primers for other TnPZ insertion sites** | |  |
|  |  |  |
| Shi117PZ-R | gaacaagtgattgctttacagcac |  |
| Shi117PZ-F | tgatttgagcgaacctagaatcagc |  |
|  |  |  |
| Shi193PZ-R | agcttgtccaaagcgtcgctc |  |
| Shi193PZ-F | caacgctaattccatgaccattaac |  |
|  |  |  |
| Shi417A-PZ Rflank | ctaggatatggcttgatttattgct |  |
| Shi417A-PZ Lflank | agcgatgaaaccatgcgagaaatc |  |
|  |  |  |
| Shi32A-PZ Rflank | tgatttcttgtatcgctacactac |  |
| Shi32A-PZ Lflank | cactaaagaagccacaaggtctaac |  |
|  |  |  |
| Shi417A-tfs3Rflank | cgaggatcttaatcgttacagacag |  |
| Shi170A-tfs3Rflank | aatcctgttgctcctcatttatgct |  |
|  |  |  |

**Table S1C. Primers used to generate ∆TnPZ and ∆tfs3 deletions**

| **Primer name** | **Sequence** | **Source** |
| --- | --- | --- |
|  |  |  |
| **TnPZ deletion** |  |  |
| RbsR2 | actaatagagcgtttggcttg | HUP-B43, left flank, 0.7kb |
| B43-L | atccacttttcaatctatatccctagatattgatttcttagaga |  |
|  |  |  |
| B43-R | cccagtttgtcgcactgataagattaaagacaccacttataacag | HUP-B43, right flank, 0.6kb |
| hp979 | tgatcatgtgcgcgttactatcat |  |
|  |  |  |
| RbsR2 | above | PeCan18B, left flank, 1kb |
| 18B-L | atccacttttcaatctatatcctctatcttgccattttaagatc |  |
|  |  |  |
| 18B-R | cccagtttgtcgcactgataagagtaagacttatagattgagtgc | PeCan18B, right flank, 0.55kb |
| hp979 | above |  |
|  |  |  |
| **tfs3 deletion** |  |  |
| J99-4 | ttactagaatgttagtttctgtctc | PeCan18B, left flank, 0.6kb |
| LJ2 | atggttcgctgggtttatcgcaaacttttctaagaaactc |  |
|  |  |  |
| RJ-1n | gatttagatgtctaaaaagcttcaattcagatgaagtcattg | PeCan18B, right flank, 0.55kb |
| RJ2 | tgctttgtgtaacttcttgt |  |
|  |  |  |
| **resistance genes** |  |  |
| C1 | gatatagattgaaaagtggat | chloramphenicol |
| C2 | ttatcagtgcgacaaactggg |  |
|  |  |  |
| kanF | gataaacccagcgaaccat | kanamycin |
| kanR | aagctttttagacatctaaatc |  |

**Table S1D. Primers used for real time PCR**

| **Primer name** | **Sequence** | **Source** |
| --- | --- | --- |
|  |  |  |
| control | gagtgctgtctccatgtttgatg | beta 2-microglobin |
|  | ctctaagttgccagccctcct |  |
|  |  |  |
| IL-8 | ctcttggcagccttcctgatt | cytokine IL-8 |
|  | tatgcactgacatctaagttctttagca |  |
