# Supplementary material for: Helicobacter Pylori's Plasticity Zones Are Novel Transposable Elements
Source: PLoS One. 2009 Sep 3;4(9):e6859. doi: 10.1371/journal.pone.0006859 (PMC2731543; doi:10.1371/journal.pone.0006859)
Supplement: Table S2 — Results of PCR based survey of plasticity zone genes in 102 H. pylori strains and H. cetorum. Sequences of PCR primers used are listed in Table S1A. The following sets of strains were tested: 22 from Japan (11 gastric cancer, 11 gastritis), 24 from urban Peru (Lima region shantytown) (12 from gastric cancer, 12 from gastritis), 24 from Spain (12 cagPAI +, 12 cagPAI −), 22 from India, 10 from Gambia and one H. cetorum (from Beluga whale). DNAs from these strains were tested with pairs of primers specific for genes in plasticity zones of reference strains 26695 and J99, and also Peruvian PeCan18B (pz32) and Japanese CPY6081 (jhp926≈, called jhp926like in GenBank Accession AY128680). Subsequent discoveries that these genes are from one of several types of transposons (TnPZs) (see text) allowed the indicated classifications: type 1 (3 pairs of primers), type 1b (7 pairs), mix for type 1/1b (1 pair), mix for type 1/type 1b/type 2 (1 pair) and type 2 (4 pairs) TnPZs. The analysis of the tfs3 component from type 2 TnPZs with 14 pairs of primers was reported previously [1]. In overview, 32% of the 102 strains yielded PCR products indicative of all three TnPZ types, 32% of two types, 27% of one type; and 8% did not yield PCR products with any primer pairs, and thus may have been TnPZ-free. The PCR data indicated that many strains contained TnPZ fragments, not complete TnPZs. Three orfs (jhp0940, jhp0947 and jhp0949), although associated with gastric pathology in some studies [2]–[5] were not significantly associated with gastric cancer in our study: jhp0940 was present in four of 12 Peruvian gastritis strains and two of 12 Peruvian gastric cancer strains; and although absent from 11 Japanese gastritis strains, was present in only one of 11 Japanese gastric cancer strains. Similarly, jhp0947, which was correlated with gastric cancer in other studies, was equally present in Peruvian gastric cancer and gastritis strains (present in four of 12 strains of each group), was absent [file pone.0006859.s002.doc]

**Table S2. Results of PCR based survey of plasticity zone genes in 102 *H. pylori* strains and *H. cetorum***

|  | *jhp* | *jhp* | *jhp* | *jhp* | *jhp* | *jhp* | *jhp* | *jhp* | *hp* | *hp* | *hp* | *jhp* | *tfs3* | *jhp* | *jhp* | *jhp* | *pz32* | *TnPZ types* |
| --- | --- | --- | --- | --- | --- | --- | --- | --- | --- | --- | --- | --- | --- | --- | --- | --- | --- | --- |
|  | *919* | *924* | *926≈* | *926* | *945* | *947* | *949* | *951* | *441* | *446* | *1000* | *928* |  | *931* | *940* | *941* |  |  |
| **strains** | *t1* | *t1* | *t1* | *1b* | *1b* | *1b* | *1b* | *1b* | *1b* | *1b* | *t1/1b* | *t1,1b,2* | *t2* | *t2* | *t2var* | *t2* | t2 |  |
|  | *tfs3a* | *tfs3a* |  |  |  |  |  |  | *tfs3b* | *tfs3b* |  |  |  |  |  |  |  |  |
| **Japan-gastritis** |  |  |  |  |  |  |  |  |  |  |  |  |  |  |  |  |  |  |
| CPY1042 | - | - | - | - | - | - | - | + | + | + | - | - | partial | - | - | - | - | 1b, 2 |
| CPY1672 | + | + | + | - | + | - | + | + | + | + | + | + | partial | - | - | - | - | 1, 1b, 2 |
| CPY2362 | + | + | + | - | + | - | + | + | + | - | + | + | no | - | - | - | - | 1, 1b |
| CPY6021 | - | - | - | - | - | - | + | + | + | + | - | + | full | + | - | - | + | 1b, 2 |
| HU29 | - | - | - | - | - | - | - | + | - | + | - | - | no | + | - | - | - | 1b, 2 |
| HU38 | - | - | - | - | - | - | - | + | + | + | - | - | no | - | - | - | - | 1b |
| HU56 | - | - | - | - | - | - | + | + | + | + | - | - | no | - | - | - | - | 1b |
| HU78 | - | - | - | - | - | - | + | + | + | + | - | - | no | - | - | - | - | 1b |
| HU87 | + | + | + | - | + | - | - | - | - | - | + | + | partial | + | - | - | + | 1, 1b, 2 |
| HU131 | + | - | + | - | + | - | - | + | + | - | + | + | no | - | - | - | - | 1, 1b |
| HU133 | + | + | - | - | + | - | - | + | - | - | + | - | partial | - | - | - | - | 1, 1b, 2 |
| **Japan-cancer** |  |  |  |  |  |  |  |  |  |  |  |  |  |  |  |  |  |  |
| HU157 | - | - | - | - | - | - | - | - | - | - | - | + | full | + | - | - | + | 2 |
| CPY6271 | + | + | + | - | + | - | - | + | + | + | + | + | no | - | - | - | - | 1, 1b |
| CPY6311 | - | - | - | - | - | - | - | + | + | + | - | - | full | + | - | - | - | 1b, 2 |
| CPY6261 | - | - | + | - | + | - | - | - | - | - | + | + | partial | - | + | - | + | 1, 1b, 2 |
| HU176 | - | - | - | - | - | - | + | + | + | + | - | - | no | - | - | - | - | 1b |
| HU178 | + | + | - | - | + | - | - | + | - | + | + | - | partial | - | - | - | - | 1, 1b, 2 |
| HU54 | - | - | - | - | - | - | - | + | + | + | - | + | full | + | - | - | + | 1b, 2 |
| HU118 | + | + | + | - | + | - | + | - | - | - | + | + | no | - | - | - | - | 1, 1b |
| HU48 | + | + | - | - | + | - | - | + | + | + | + | - | no | - | - | - | - | 1, 1b |
| HU71 | - | - | - | - | - | - | + | + | + | + | - | - | no | - | - | - | - | 1b |
| CPY6081 | + | + | + | - | + | - | + | + | + | + | + | + | no | - | - | - | - | 1, 1b |
| **Peru-cancer** |  |  |  |  |  |  |  |  |  |  |  |  |  |  |  |  |  |  |
| PeCan1B | + | + | - | - | - | - | - | + | - | - | - | - | no | - | - | - | - | 1, 1b |
| PeCan2A | + | + | + | - | + | - | - | - | - | - | - | + | no | - | - | - | - | 1, 1b |
| PeCan4A | - | - | + | - | + | + | + | + | + | - | + | + | no | - | - | - | - | 1, 1b |
| PeCan9A | + | - | - | - | + | + | + | + | - | - | + | + | no | - | - | + | - | 1, 1b, 2 |
| PeCan10B | + | - | - | + | + | + | + | + | - | - | + | + | full | + | - | + | - | 1, 1b, 2 |
| PeCan14B | - | - | - | - | - | - | - | - | - | - | - | - | partial | - | + | - | + | 2 |
| PeCan16A | - | - | - | - | - | - | - | - | - | - | - | - | no | - | - | - | - | no |
| PeCan18B | - | - | - | - | - | - | + | + | - | - | - | + | full | + | - | + | + | 1b, 2 |
| PeCan22A | + | - | - | - | - | + | + | + | - | - | - | - | partial | + | - | - | + | 1, 1b, 2 |
| PeCan28B | + | + | - | - | + | - | - | - | - | - | + | - | no | + | + | - | + | 1, 1b, 2 |
| PeCan32A | + | + | + | - | + | - | - | - | - | - | + | - | no | + | - | - | - | 1, 1b, 2 |
| PeCan38B | + | - | + | - | + | - | - | - | - | - | + | + | partial | - | - | - | + | 1, 1b, 2 |
| **Peru-gastritis** |  |  |  |  |  |  |  |  |  |  |  |  |  |  |  |  |  |  |
| SJM9 | + | + | - | - | - | - | - | + | - | - | - | - | partial | + | - | + | + | 1, 1b, 2 |
| SJM26 s.c. | - | - | - | - | - | - | - | - | - | - | - | - | no | - | - | - | - | no |
| SJM42 s.c. | - | - | - | - | - | - | - | - | - | - | - | - | no | - | + | - | + | 2 |
| SJM68 s.c. | + | + | - | - | - | - | - | + | - | - | - | - | no | - | - | - | - | 1, 1b |
| SJM75 s.c. | + | - | - | - | - | + | + | + | - | - | - | - | no | - | - | - | - | 1, 1b |
| SJM79 s.c. | - | - | - | - | - | - | - | + | + | - | - | - | no | - | - | - | - | 1b |
| SJM91 s.c. | + | - | - | - | - | - | - | + | - | - | - | - | partial | - | + | + | + | 1, 1b, 2 |
| SJM148 s.c. | - | - | - | + | + | + | + | + | - | - | + | + | full | + | - | + | + | 1, 1b, 2 |
| SJM179 b.c. | - | - | - | - | - | - | - | + | + | - | - | - | no | - | - | - | - | 1b |
| SJM180A | + | + | + | - | + | + | + | + | - | - | - | + | partial | + | + | + | + | 1, 1b, 2 |
| SJM184A | + | - | - | - | + | + | + | - | - | - | + | + | full | + | + | + | + | 1, 1b, 2 |
| SJM189A | - | - | - | - | + | - | + | - | - | - | - | - | no | + | - | - | - | 1b, 2 |
| **Spain *cag* PAI+** |  |  |  |  |  |  |  |  |  |  |  |  |  |  |  |  |  |  |
| HUP-B41 | - | + | - | + | + | + | + | + | + | + | - | - | full | + | - | + | + | 1, 1b, 2 |
| HUP-B49 | + | + | - | + | + | + | + | + | - | - | - | + | no | - | - | - | - | 1, 1b |
| HUP-B50 | - | - | + | - | + | + | + | + | - | - | - | + | no | + | - | + | - | 1, 1b, 2 |
| HUP-B51 | - | - | - | - | - | - | - | - | - | - | - | + | no | + | - | + | + | 2 |
| HUP-B58 | + | - | + | - | + | + | + | + | - | - | + | + | no | + | - | - | + | 1, 1b, 2 |
| HUP-B59 | + | + | + | - | - | - | - | - | - | - | - | + | no | - | - | + | - | 1, 2 |
| HUP-B62 | - | - | - | - | + | + | + | + | - | - | - | - | full | + | - | + | + | 1b, 2 |
| HUP-B63 | - | - | - | - | - | - | - | - | - | - | - | - | no | + | - | + | - | 2 |
| HUP-B71 | - | - | - | - | - | - | - | + | - | - | - | - | no | - | - | - | - | 1b |
| HUP-B79 | - | - | - | + | + | + | + | + | - | + | + | + | no | - | - | + | - | 1b, 2 |
| HUP-B85 | - | - | - | - | - | - | - | - | - | - | - | - | full | + | - | + | + | 2 |
| HUP-B86 | - | - | - | - | - | - | - | - | - | - | - | - | no | - | - | - | - | no |
| **Spain cag PAI-** |  |  |  |  |  |  |  |  |  |  |  |  |  |  |  |  |  |  |
| HUP-B43 | - | - | - | + | - | - | - | + | - | + | - | - | no | - | - | - | - | 1b |
| HUP-B45 | - | - | - | - | - | - | - | + | + | + | + | + | no | - | - | + | - | 1b, 2 |
| HUP-B46 | + | + | - | + | + | + | + | + | + | - | + | + | partial | - | - | + | + | 1, 1b, 2 |
| HUP-B53 | - | - | - | - | - | - | - | - | - | - | - | - | no | - | - | - | - | no |
| HUP-B56 | - | - | - | - | - | - | - | - | - | - | - | + | no | - | - | - | - | 2? |
| HUP-B60 | - | - | - | + | + | + | + | + | + | + | + | + | no | - | - | - | - | 1b |
| HUP-B65 | - | - | - | - | - | - | - | - | - | - | - | + | full | + | - | + | + | 2 |
| HUP-B70 | - | - | + | - | + | + | + | + | + | + | - | + | no | - | - | - | - | 1, 1b |
| HUP-B76 | - | - | + | - | + | - | - | - | - | - | + | + | no | - | - | - | - | 1, 1b |
| HUP-B80 | - | - | - | - | - | - | - | - | - | + | - | - | no | - | - | - | - | 1b? |
| HUP-B82 | - | - | - | - | - | - | - | - | - | - | - | + | full | + | - | + | + | 2 |
| HUP-B84 | - | - | - | - | - | - | - | - | - | - | - | + | full | + | - | + | + | 2 |
| **India** |  |  |  |  |  |  |  |  |  |  |  |  |  |  |  |  |  |  |
| I-7A | - | - | - | - | - | - | - | + | - | - | - | + | full | + | - | - | - | 1b, 2 |
| I-27B s.c. | - | - | - | - | - | - | - | - | - | - | - | - | no | - | - | - | - | no |
| I-34B s.c. | - | - | - | - | - | - | - | - | - | - | - | - | partial | + | - | - | - | 2 |
| I-39A b.c. | - | - | + | - | - | + | + | + | - | - | + | + | no | - | - | - | - | 1, 1b |
| I-44A | - | - | + | - | + | + | + | + | - | - | + | + | no | - | - | - | - | 1, 1b |
| I-48B | + | - | + | - | + | - | - | - | - | - | + | + | no | - | - | - | - | 1, 1b |
| I-49 | - | - | + | - | + | - | - | - | - | - | + | + | partial | - | - | - | + | 1, 1b, 2 |
| I-56B b.c. | - | - | - | - | - | - | - | - | - | - | - | + | full | + | - | - | - | 2 |
| I-66B s.c. | - | - | - | - | - | - | - | + | - | - | + | - | no | - | - | - | - | 1b |
| I-67B | - | - | + | - | + | + | + | + | - | - | - | + | no | - | - | - | - | 1, 1b |
| I-75A s.c. | - | - | + | - | + | - | - | - | - | - | - | + | full | + | - | + | - | 1, 1b, 2 |
| I-77A | - | - | + | - | + | - | - | + | - | - | - | + | partial | + | - | - | - | 1, 1b, 2 |
| Santal10 | - | - | - | - | - | - | - | - | - | - | - | - | no | - | - | - | - | no |
| Santal31 | - | - | + | - | + | - | - | - | - | - | + | + | partial | + | - | - | - | 1, 1b, 2 |
| Santal49 | + | + | + | - | + | - | - | - | - | - | + | + | no | - | - | - | - | 1a, 1b |
| Santal51 | - | - | - | - | - | - | - | - | - | - | - | - | no | - | - | - | - | no |
| Santal52 | - | - | + | - | + | - | - | + | - | - | - | + | no | + | + | - | + | 1, 1b, 2 |
| Santal54 | - | - | + | - | + | - | - | - | - | - | + | + | partial | - | - | + | - | 1, 1b, 2 |
| Chen1 | - | - | - | - | - | - | - | - | - | - | - | + | partial | + | - | - | - | 2 |
| Chen2 | - | - | - | + | + | + | + | + | - | - | - | + | no | - | - | - | - | 1b |
| Chen3 | - | - | - | + | + | + | + | + | - | - | + | + | no | - | - | - | - | 1b |
| Chen4 | - | - | - | + | + | + | + | + | - | + | + | + | no | - | - | - | - | 1b |
| **Gambia** |  |  |  |  |  |  |  |  |  |  |  |  |  |  |  |  |  |  |
| 94/24 | + | + | - | + | + | + | + | + | - | - | + | + | full | + | - | + | + | 1, 1b, 2 |
| HP28B | + | + | + | - | + | + | - | - | - | - | + | + | no | + | - | - | + | 1, 1b, 2 |
| 94/64A | + | + | + | - | + | + | + | + | - | - | + | + | no | - | - | + | + | 1, 1b, 2 |
| 9419 | + | + | + | - | + | + | + | + | - | - | + | + | full | + | - | + | + | 1, 1b, 2 |
| 4655/1 | - | - | - | - | - | - | - | - | - | - | - | - | no | - | - | - | - | no |
| 4797/3 | + | + | - | + | + | + | + | + | - | - | + | + | full | + | - | + | + | 1, 2, 1b |
| 4842-2 | + | + | + | - | + | + | + | + | - | - | - | + | full | + | - | + | + | 1, 2, 1b |
| G012 I | - | - | - | - | - | + | + | + | - | - | - | + | full | + | - | + | + | 1b, 2 |
| JS004 II | + | + | - | + | + | - | + | + | - | - | + | - | full | + | - | - | - | 1, 1b, 2 |
| HP24X III | + | + | - | - | + | + | + | + | - | - | - | + | no | - | - | - | - | 1, 1b |
| *H. cetorum* | - | - | - | - | - | - | - | - | - | - | - | - | full | + | + | + | - | 2 |

Sequences of PCR primers used are listed in Table S1A. The following sets of strains were tested: 22 from Japan (11 gastric cancer, 11 gastritis), 24 from urban Peru (Lima region shantytown) (12 from gastric cancer, 12 from gastritis), 24 from Spain (12 *cagPAI*^+^, 12 *cagPAI*^-^), 22 from India, 10 from Gambia and one *H. cetorum* (from Beluga whale). DNAs from these strains were tested with pairs of primers specific for genes in plasticity zones of reference strains 26695 and J99, and also Peruvian PeCan18B (*pz32*) and Japanese CPY6081 (*jhp926≈*, called *jhp926like* in GenBank Accession AY128680). Subsequent discoveries that these genes are from one of several types of transposons (TnPZs) (see text) allowed the indicated classifications: type 1 (3 pairs of primers), type 1b (7 pairs), mix for type 1/1b (1 pair), mix for type 1/type 1b/type 2 (1 pair) and type 2 (4 pairs) TnPZs. The analysis of the *tfs3* component from type 2 TnPZs with 14 pairs of primers was reported previously [1]. In overview, 32% of the 102 strains yielded PCR products indicative of all three TnPZ types, 32% of two types, 27% of one type; and 8% did not yield PCR products with any primer pairs, and thus may have been TnPZ-free. The PCR data indicated that many strains contained TnPZ fragments, not complete TnPZs.

Three orfs (*jhp0940, jhp0947* and *jhp0949*), although associated with gastric pathology in some studies [2-5] were not significantly associated with gastric cancer in our study: *jhp0940* was present in four of 12 Peruvian gastritis strains and two of 12 Peruvian gastric cancer strains; and although absent from 11 Japanese gastritis strains, was present in only one of 11 Japanese gastric cancer strains. Similarly, *jhp0947*, which was correlated with gastric cancer in other studies, was equally present in Peruvian gastric cancer and gastritis strains (present in four of 12 strains of each group), was absent from Japanese strains. *jhp0949* was present in four of 11 Japanese gastric cancer and five of 11 gastritis strains and in five of 12 Peruvian gastric cancer as well as gastritis strains.
