# Supplementary figures and images for: Helicobacter Pylori's Plasticity Zones Are Novel Transposable Elements
Source: PLoS One. 2009 Sep 3;4(9):e6859. doi: 10.1371/journal.pone.0006859 (PMC2731543; doi:10.1371/journal.pone.0006859)

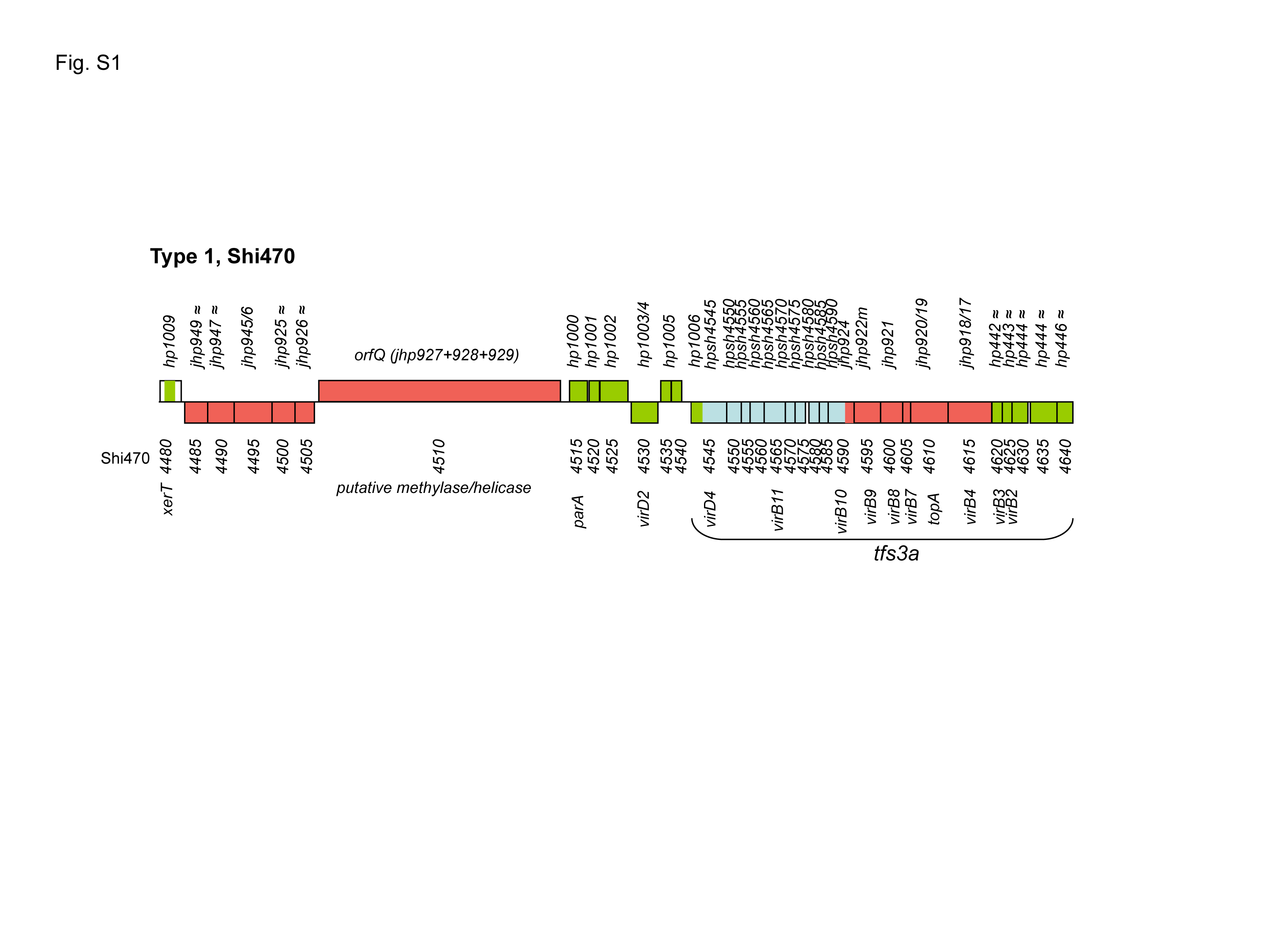

Supplement: Figure S1 — Type 1 TnPZ in Peruvian Amazon Shimaa village strain Shi470. This TnPZ is 39 kb long, has 33 orfs and is inserted into the homolog of strain 26695 gene hp0488 (TnPZ coordinates 874704–913876 bp, orfs hpsh_04480 - hpsh_04640 in this strain's full genome sequence; GenBank Accession CP001072). It has a novel 5.8 kb segment, not represented in strains 26695 or J99 (hpsh_4550 through 3′ half of hpsh_4590), but which is present in other type 1 TnPZs and in the type 1b TnPZ of German strain P12. The Shi470 type 1 TnPZ contains a type IV protein secretion system gene cluster (hpsh_04545 - hpsh_4640) that is similar in size (16.3 kb), gene content and arrangement to tfs3 in type 2 TnPZs [1], but with very low, if any, DNA sequence identity to them. It was therefore designated tfs3a. Genes in tfs3a that resemble those in canonical type IV secretion systems are: virD4 (hpsh_04545), virB11 (hpsh_04565), virB9 (hpsh_04595), virB8 (hpsh_04600), virB7 (hpsh_04605), virB4 (hpsh_04615), virB3 (hpsh_04620), virB2 (hpsh_04625). Symbols. Boxes above the line, orfs transcribed rightward; below the line, transcribed leftward. Box color coding: green, TnPZ orfs with homologies to reference strain 26695 (orf numbers start with hp); red, TnPZ orfs with homologies to reference strain J99 (orf numbers start with jhp). Orfs marked with ≈ have relatively low protein level identity (35–70%) to their homologs in reference strains 26695 and J99; protein level identities of other orfs to those of one or the other reference strains range from 90–95%. Boxes in light blue - Shi470 strain specific orfs, not found in strains 26695 or J99. Above the line, homologies to reference strains; numbers below the line, orf designations as in GenBank annotation (preceded by hpsh_). (1.16 MB TIF) [file pone.0006859.s003.tif]

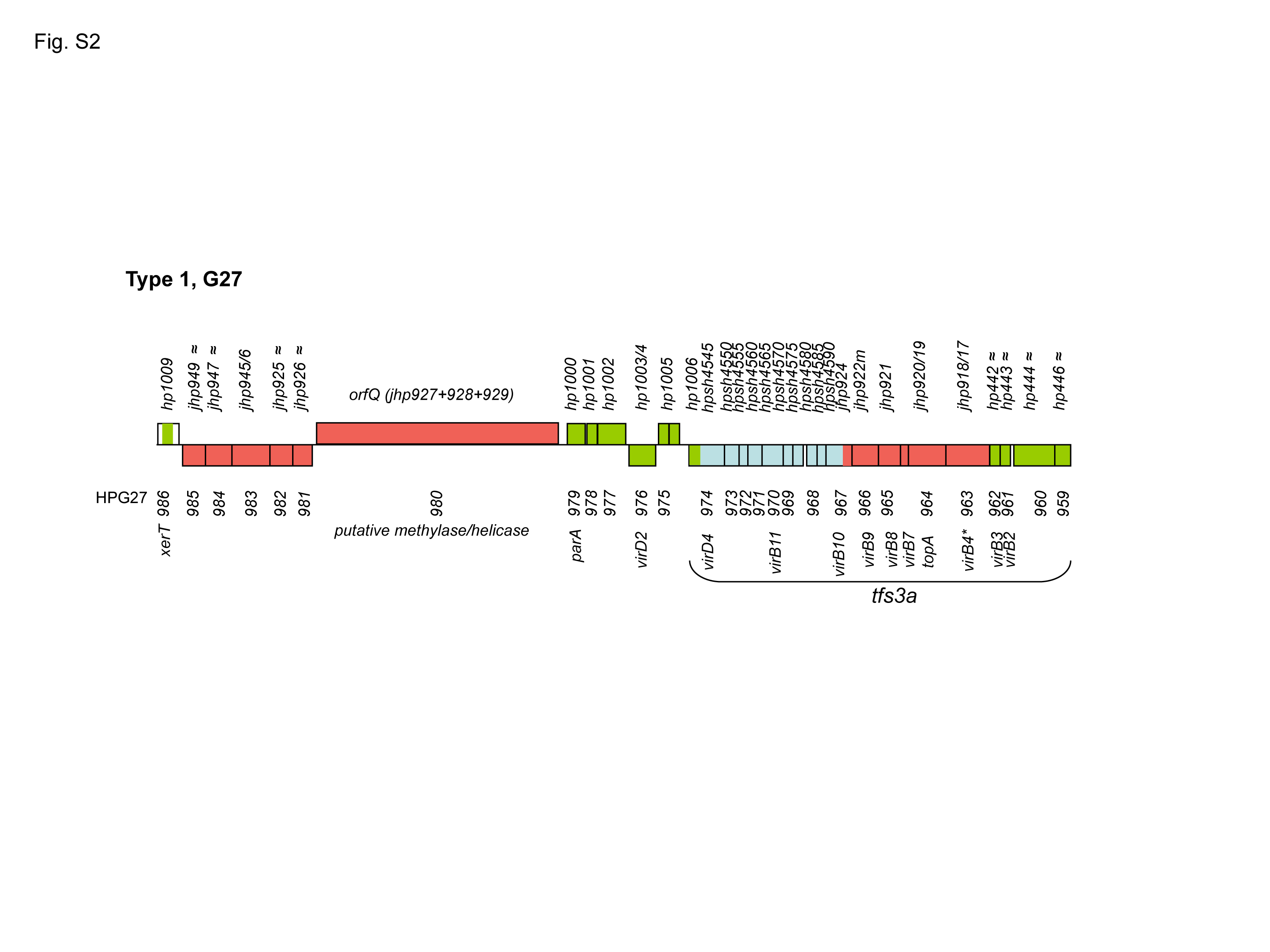

Supplement: Figure S2 — Type 1 TnPZ in the genome sequence of Italian strain G27. This 39 kb element (coordinates 1045701–1085075 bp in strain G27 genome sequence of Baltrus et al.; GenBank Accession CP001173) is very similar to the type 1 TnPZ in strain Shi470 (95% DNA identity across its length without gaps). Numbers below the line (986 through 959), orf designations in strain G27 GenBank annotation (preceded by HPG27_). The DNA sequences of some of the TnPZ orfs annotated in Shi470 but not annotated in G27, are nevertheless present (hpsh_04605, hpsh_04585, hpsh_04575, hpsh_04540). if a gene is predicted to be inactive (due to a stop codon or frameshift), it is marked with an asterisk (*). For other symbols, see legend to Fig. S1. (1.10 MB TIF) [file pone.0006859.s004.tif]

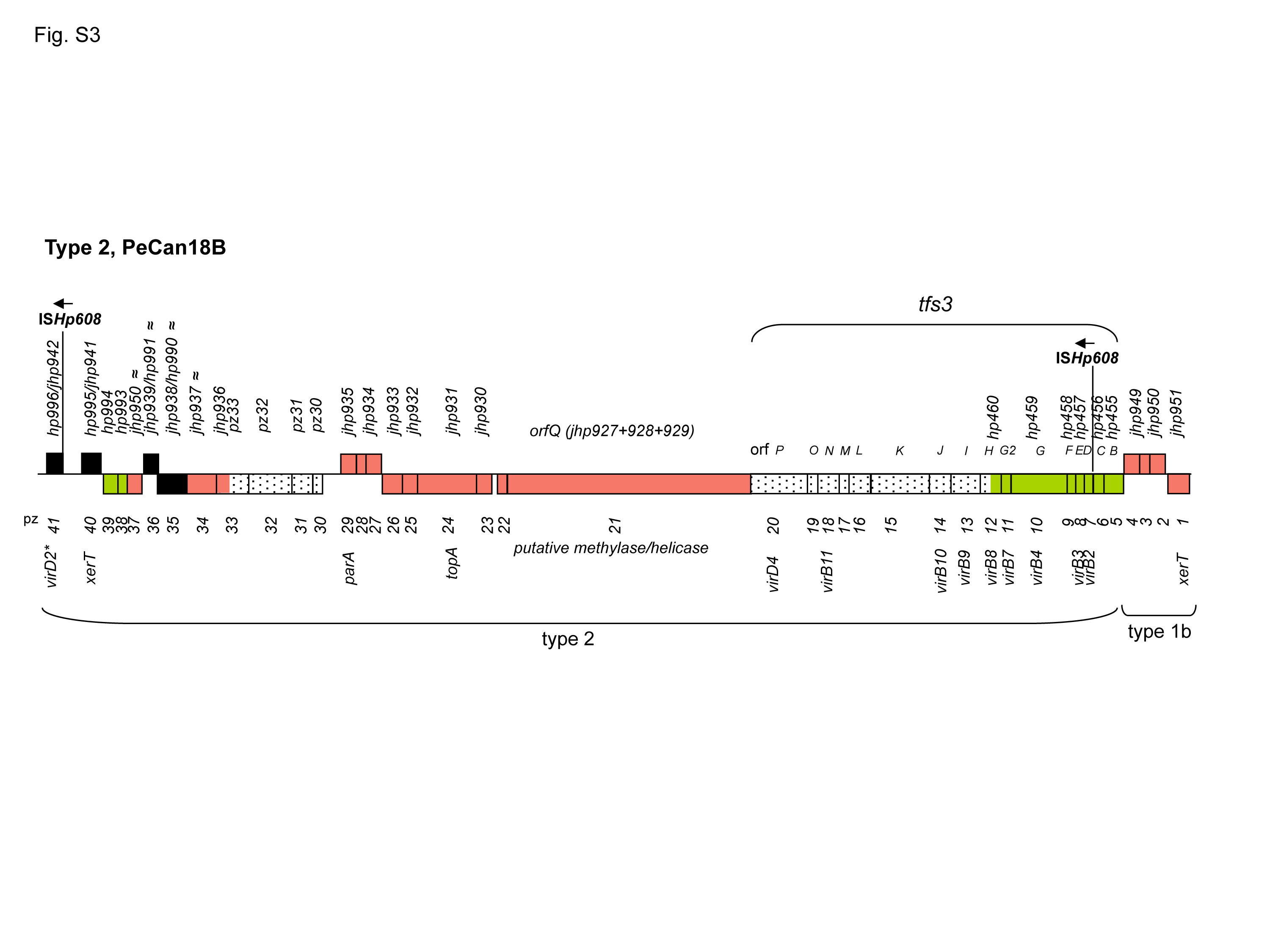

Supplement: Figure S3 — Type 2 TnPZ and type 1b TnPZ remnant in urban Peruvian strain PeCan18B. The segment containing TnPZ sequences in PeCan18B is 52.4 kb long (coordinates 864–53286 bp in GenBank Accession AF487344; genes also designated pz1 through pz41;pz gene numbers below line), and includes two segments of novel DNA, not known from reference strains 26695 or J99 (in dots): 10.8 kb in its tfs3 component (3′-end of orfH through orfP) and 4.7 kb of additional sequence (pz30 through 5′-end of pz33). These sequences are also present in other full size type 2 TnPZs. The type 2 TnPZ in PeCan18B extends from the genes designated pz5 - pz41, and is inserted next to a remnant of a type 1b TnPZ (genes pz1 - pz4, homologs of jhp0951 - jhp0949). This structure is inferred to have resulted from type 2 TnPZ insertion into a resident type 1b element, and deletion adjacent to one type 2 TnPZ end. Sites of ISHp608 element insertion and their orientations of are indicated. Boxes in dots, orfs originally found in PeCan18B (tfs3 orfs H - P, pz30-pz33), but present in all full size type 2 TnPZs. TnPZ orfs with homologies genes found in both 26695 and J99 are indicated in black. For other symbols, see legends to Figs. S1 and S2. (1.14 MB TIF) [file pone.0006859.s005.tif]

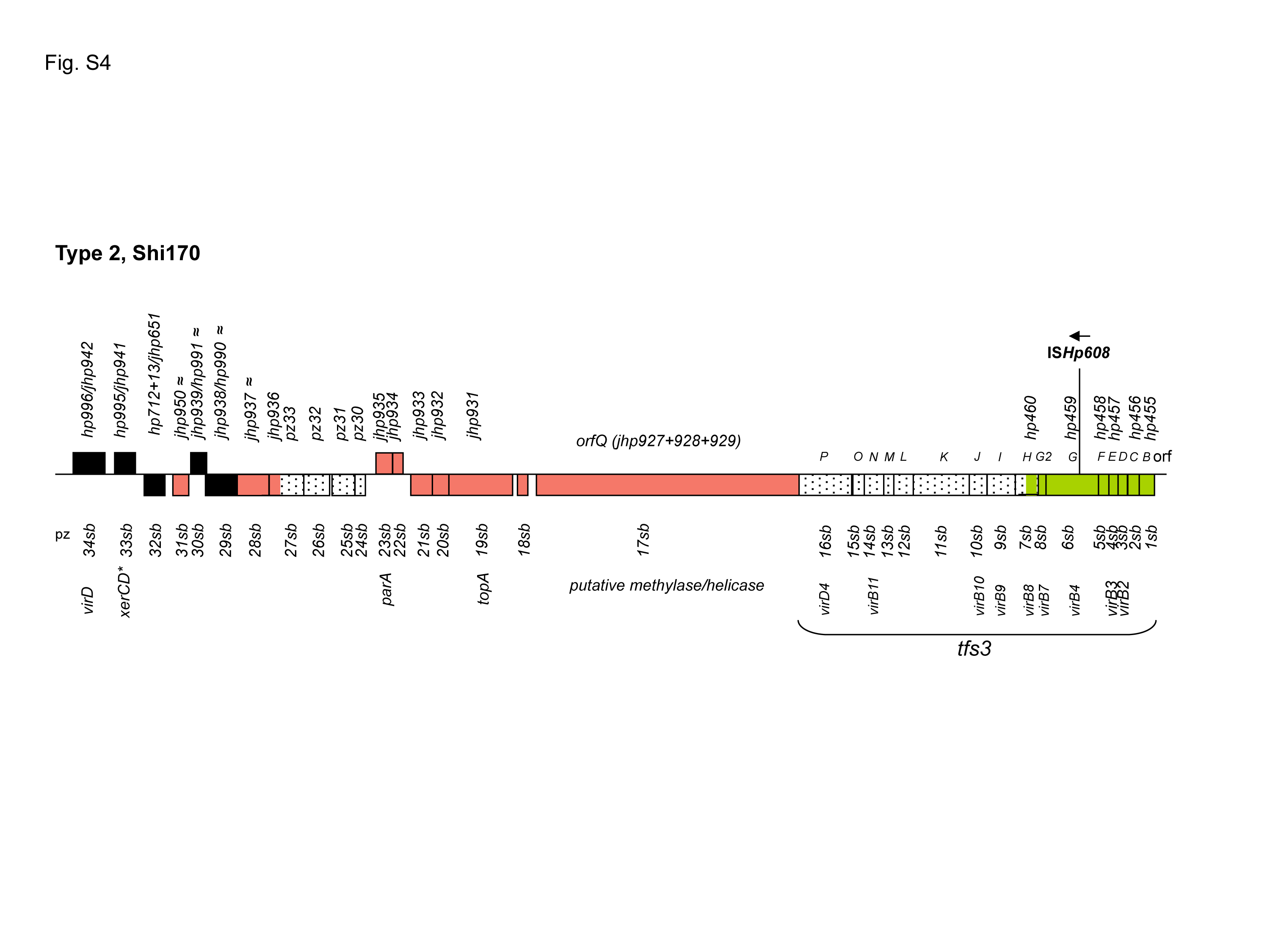

Supplement: Figure S4 — Type 2 TnPZ in Peruvian Amazon Shimaa village strain Shi170. This 46.5 kb long TnPZ has 34 orfs, 16 of which belong to tfs3 (TnPZ coordinates 574–47093 bp in GenBank Accession EU807988). It is similar to the type 2 TnPZ in PeCan18B in gene content and arrangement, except that the hp0993 and hp0944 homologs in PeCan18B were replaced by homologs of hp0712 and hp0713, which are not within the plasticity zones of reference strains J99 or 26695. This TnPZ is inserted between homologs of J99 genes jhp0210 and jhp0211. Shi170 also contains a type 1 TnPZ that is closely related by PCR criteria to the sequenced element in Shi470. For symbols, see legends to Figs. S1 - S3. (1.12 MB TIF) [file pone.0006859.s006.tif]

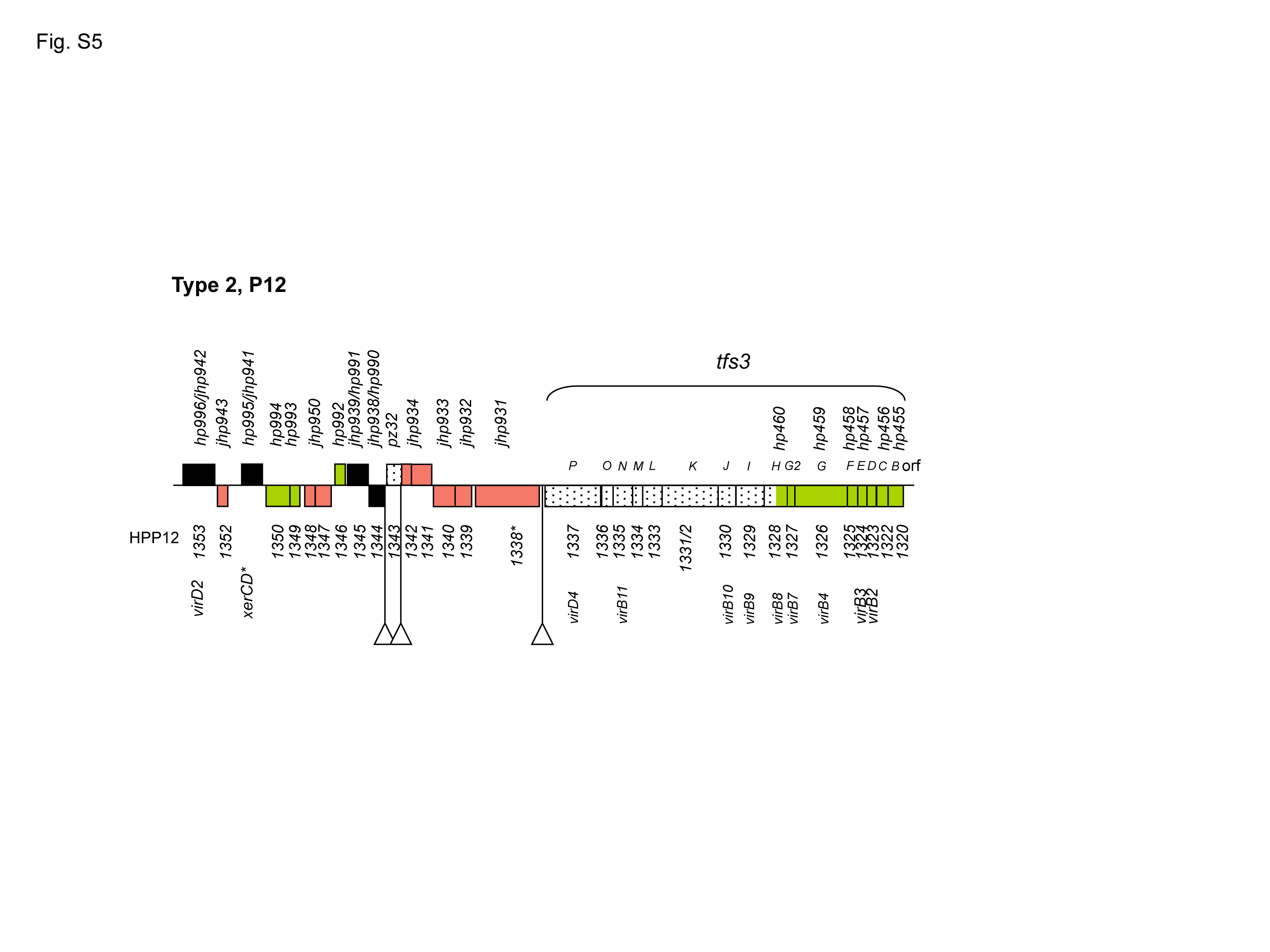

Supplement: Figure S5 — Type 2 TnPZ in German strain P12. This TnPZ is just 30 kb long (coordinates 1394773–1424783 bp in strain P12 genome sequence of Fischer et al. GenBank Accession CP001217), has 32 annotated orfs (HPP12_1320 - HPP12_1353) and contains three deletions relative to other type 2 TnPZ elements such as that in PeCan18B (absence of pz21 - pz23, pz29 - pz31 and pz33 - pz34, Fig. S3). This type 2 TnPZ is inserted into a homolog of jhp1272 (putative) and co-exists in this strain with a full length type 1b TnPZ. Sites of deletion are marked with open triangles; for other symbols, see legends to Figs. S1 - S3. (1.04 MB TIF) [file pone.0006859.s007.tif]

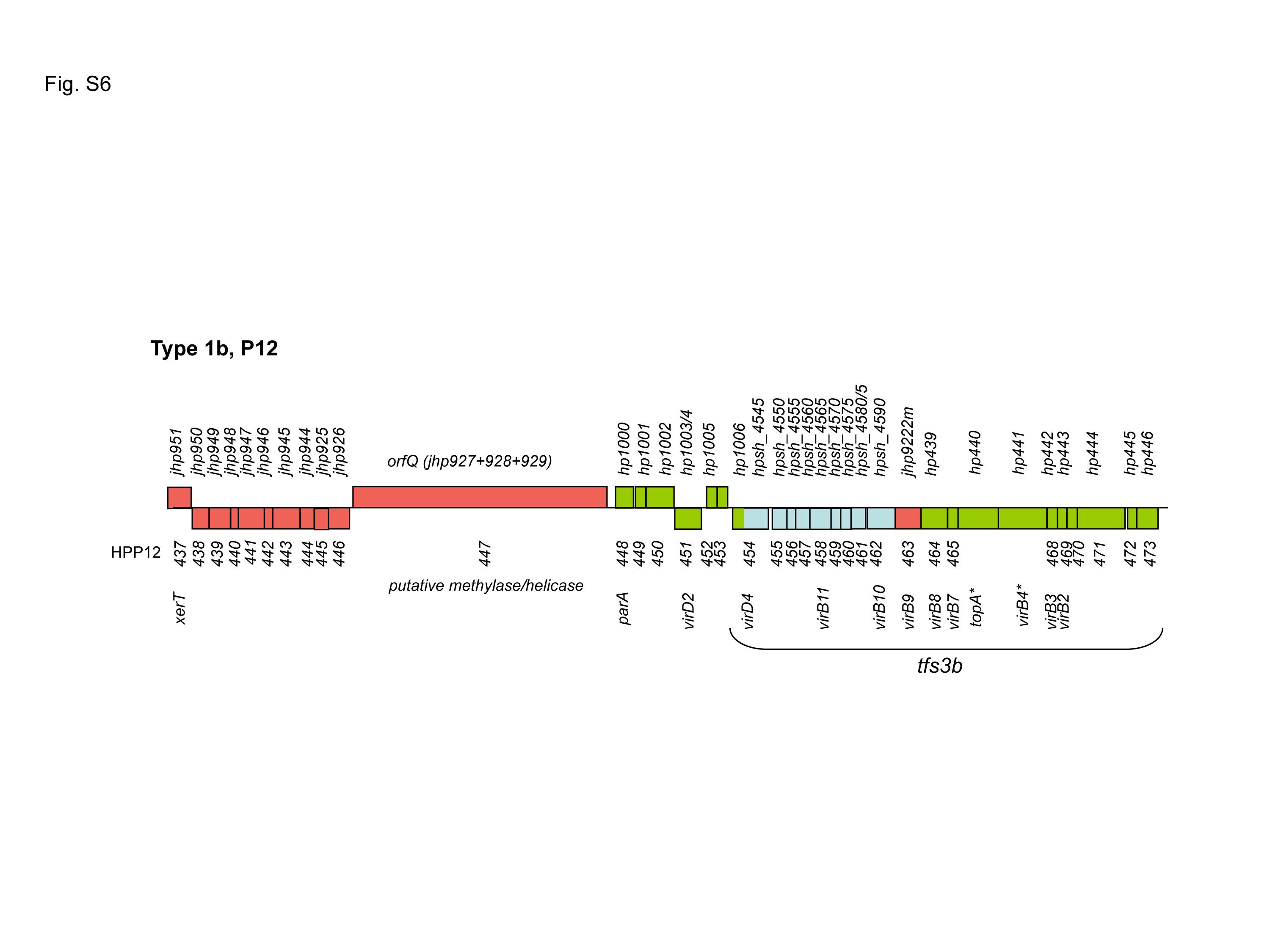

Supplement: Figure S6 — Type 1b TnPZ in German strain P12. This TnPZ is 40.8 kb long (coordinates 452020–492773 bp in GenBank Accession CP001217 of Fischer et al), has 35 orfs (HPP12_0437 - HPP12_0473) and is inserted into a homolog of hp0464 (hsdR) of strain 26695. This type 1b TnPZ is similar in gene organization to type 1 TnPZs found in strains Shi470 (Fig. S1) and G27 (Fig. S2), although it differs markedly from them in sequence for ∼8 kb from each end. Included among the divergent genes are xerCD on the left, and the virB8 - virB2 segment of the type IV secretion gene cluster, here called tfs3b. The topA and virB4 genes contain inactivating mutations and thus are pseudogenes. For symbols, see legends to Figs. S1- S3 and S5. (1.13 MB TIF) [file pone.0006859.s008.tif]

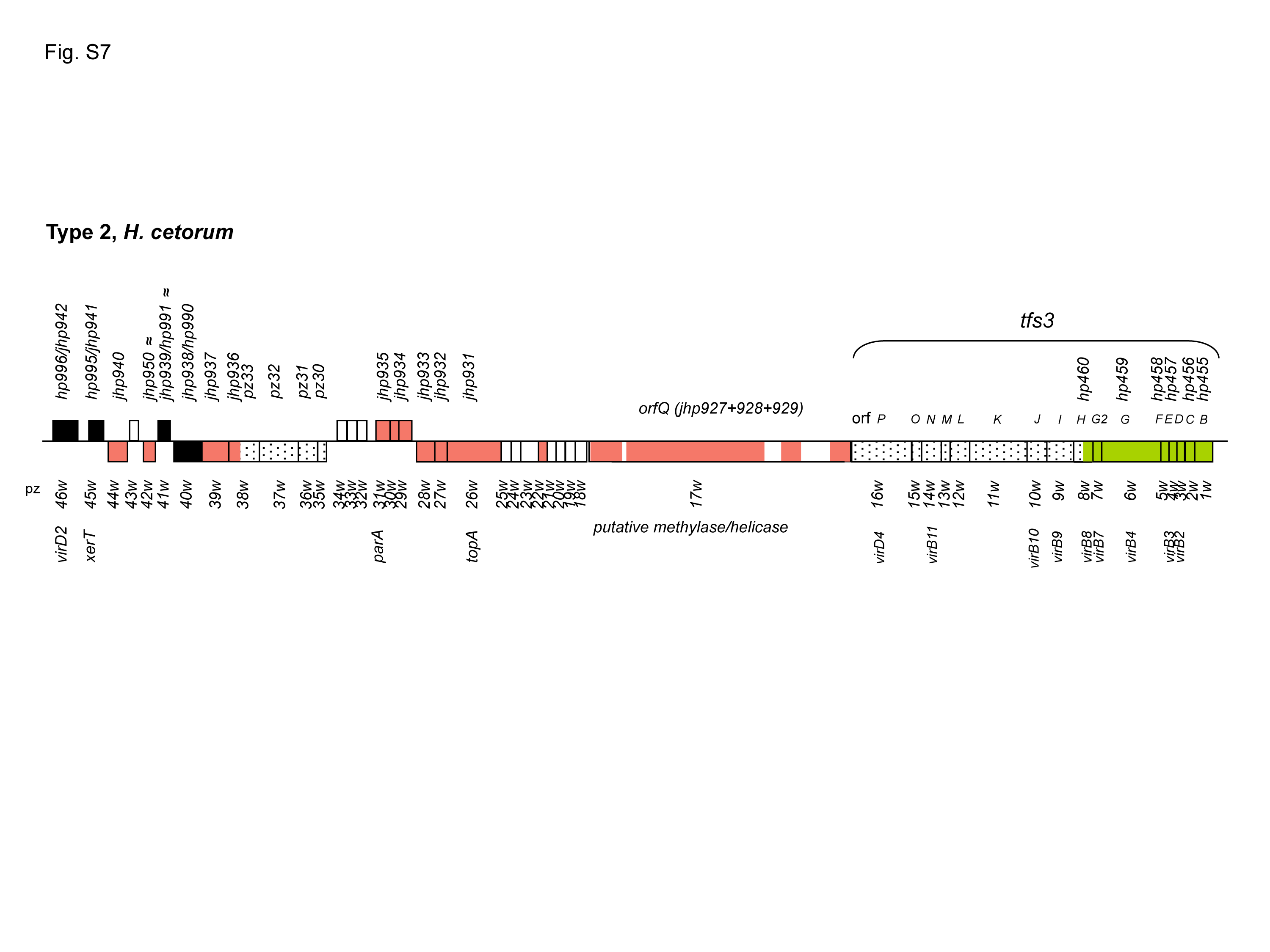

Supplement: Figure S7 — Type 2 TnPZ in H. cetorum strain MIT-00-7128. This TnPZ is 55 kb long (coordinates 3698–58762 bp in GenBank Accession EU015081) and has 46 orfs, 16 of them belong to tfs3. It is inserted in a strain-specific region, but close to homologs of restriction-modification genes on one side and a GTP-binding protein gene on the other (hp0303/jhp0288). Although this H. cetorum strain was cultured from a Beluga whale, its TnPZ had a striking resemblance to the type 2 TnPZs of H. pylori (PeCan18B, Shi170) based on sequence homology and gene arrangement. Nevertheless, it is also unique in containing 8.5 kb sequences not found in other known TnPZs (in white). 3.5 kb of unique DNA is distributed among three parts of the huge orfQ (putative helicase/methyltransferase) gene (4043 codons, pz17w, vs. 2879 codons, pz21, in PeCan18B). The other 5 kb is scattered among several clusters of short orfs (pz18w - pz21w, pz22w - pz25w, pz32w -pz34w, pz43w). For symbols, see legends to Figs. S1- S3 and S5. (1.11 MB TIF) [file pone.0006859.s009.tif]

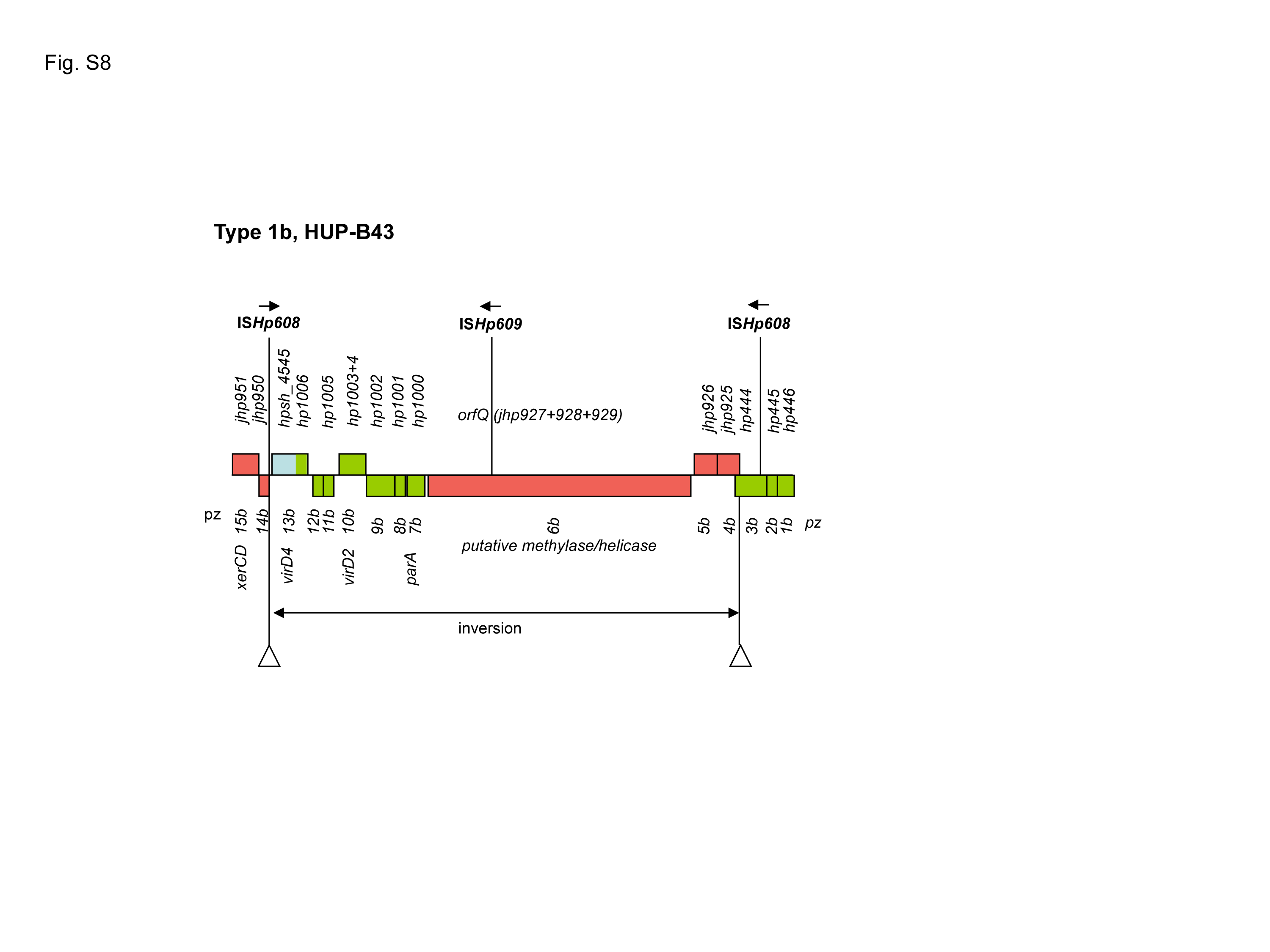

Supplement: Figure S8 — Type 1b TnPZ in Spanish strain HUP-B43. This TnPZ is just 27.4 kb long (coordinates 1285–28713 bp in GenBank Accession AY487825), and has 21 orfs, two ISHp608s and one ISHp609. It has a complete virD4 homolog (pz13b) whose 3′-end closely resembles a truncated version in the strain 26695 genome (hp1006). This TnPZ has a large internal inversion (pz4b - pz13b) relative to the type 1b TnPZ of strain P12 (orfs HPP12_0439 - HPP12_0444 on the left; orfs HPP12_0455 - HPP12_0470 on the right) with multigene deletions at each inversion breakpoint. One inversion/deletion breakpoint coincides with an ISHp608 insertion (pz14b, truncated version of jhp0950). No IS element is precisely at the other breakpoint, although one might have been present earlier and then become lost by subsequent deletion. The insertion of ISHp608 in pz3b is not associated with any deletion. For symbols, see legends to Figs. S1- S3 and S5. (0.91 MB TIF) [file pone.0006859.s010.tif]

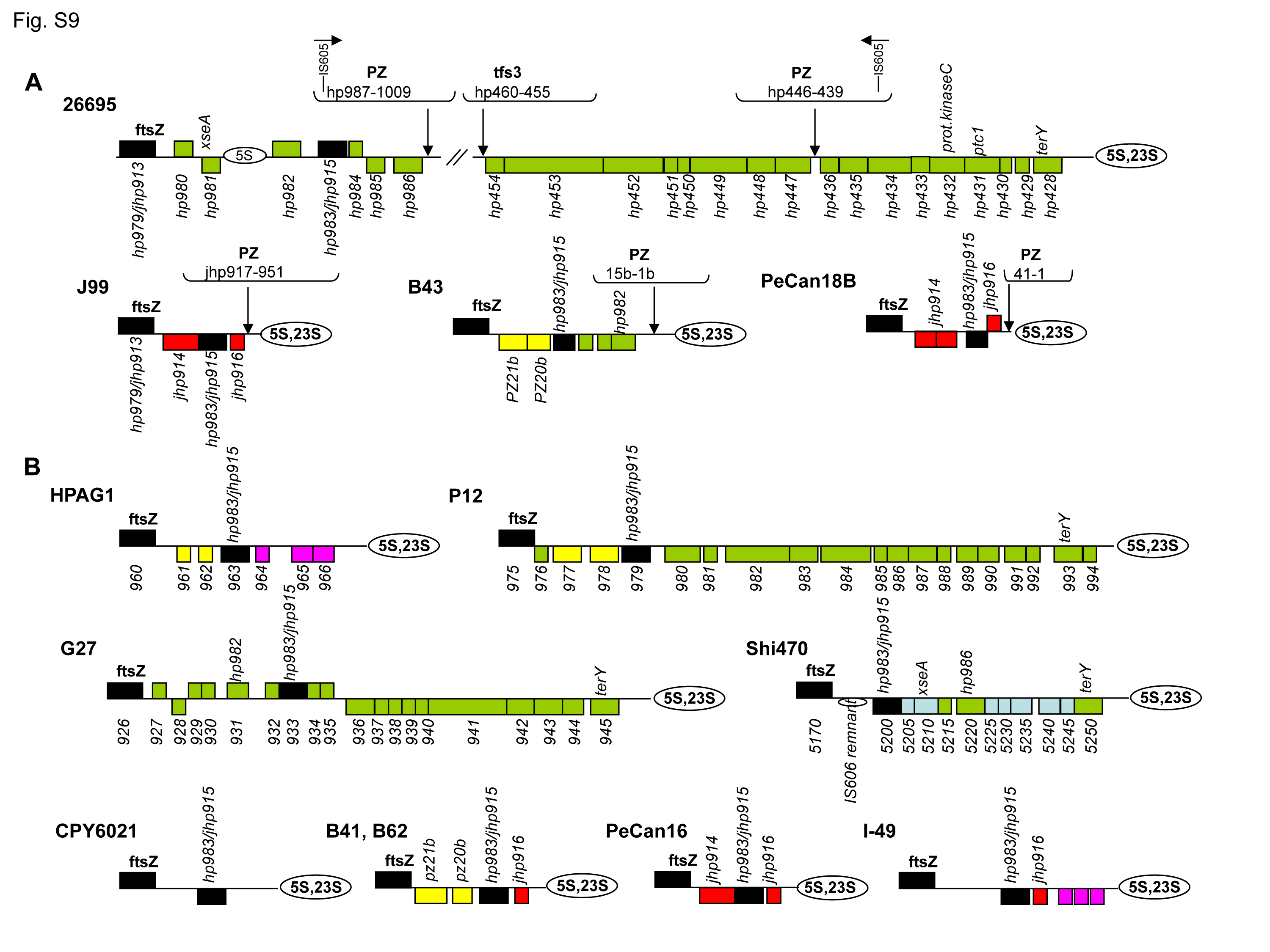

Supplement: Figure S9 — Region between ftsZ gene and the 5S-23S rRNA locus. Color coding as in Figs. S1- S8; other colors represent strain specific sequences, sometimes shared by other strains but absent in the 26695 or J99 genome sequences. This region has been used frequently for TnPZ insertion. A. Region between ftsZ and 5S-23S rRNA without TnPZ insertion (“empty site”), where TnPZ is inserted in other genomic locations or is absent (HPAG1, PeCan16A). Seven strains were PCR amplified with primers located in ftsZ and 5SrRNA (979+5S, PCR size 1.6 to 4.6 kb, Table S1A) and sequenced (GenBank Accession EU019081 - EU019087), five of which are presented here (Japanese CPY6021, 2.3 kb; Spanish HUP-B41 and B62, 3.6 kb; urban Peruvian PeCan16A, 3.6 kb and Indian I-49, 4.2 kb). Other sequences were taken from genome annotations (HPAG1, 4 kb; P12, 14 kb; G27, 13 kb and Shi470, 7 kb). Shi470 has a remnant of IS606 (orfA absent, orfB with deletion) next to ftsZ. Orf numbers below in HPAG1, P12, G27 and Shi470 refer to those used in genome annotations. B. Region between ftsZ and 5S,23S rRNA with sites of insertion of TnPZ remnants marked by arrows in strains 26695 (in total 20 kb), J99 (3.6 kb), HUP-B43 (4.5 kb) and PeCan18B (4 kb). (1.78 MB TIF) [file pone.0006859.s011.tif]

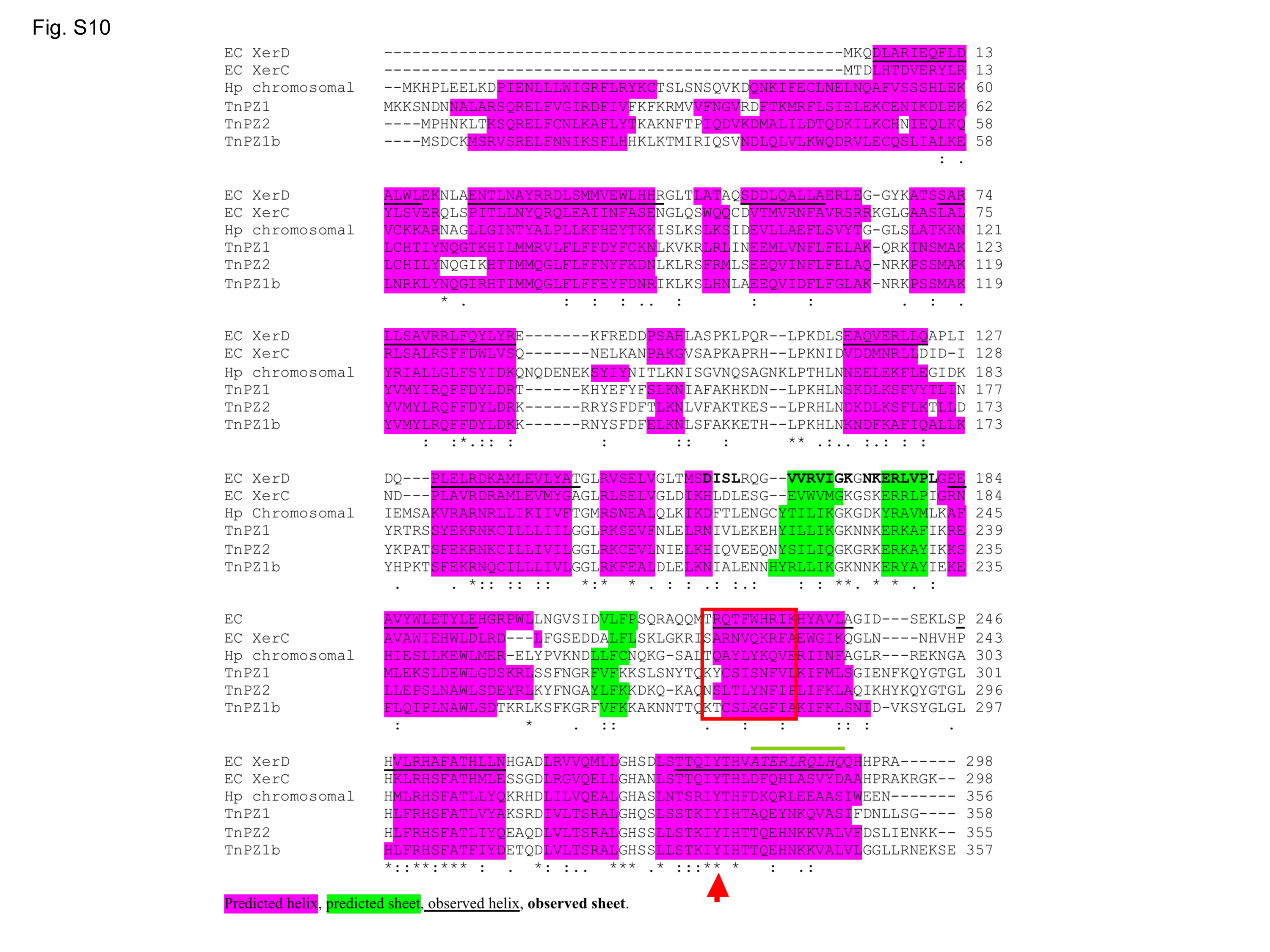

Supplement: Figure S10 — Structural predictions and comparisons for XerCD-like proteins. This multiple sequence alignment was created for XerT proteins encoded by H. pylori TnPZ types 1, 2 and 1b (from strains Shi470, PeCan18B and P12, respectively), chromosomal XerH from Shi470 strain (HPSH_03490) and the XerD and XerC proteins of E. coli, using ClustalW. The alignment was validated and slightly refined based on comparing secondary structures predicted by PSI-PRED algorithms (http://bioinf.cs.ucl.ac.uk/psipred/). The observed E. coli XerD secondary structure (purple, predicted alpha helix; underline, observed alpha helix; green, predicted beta sheet; bold, observed beta sheet) is also depicted on the figure [6]. Note that regardless of the lack of sequence similarity in the N-terminal domain, the secondary structures align well throughout the sequences. This suggests that all four H. pylori proteins have folds similar to that of E. coli XerD. Boxed sequence indicates residues implicated in DNA binding for the E. coli XerD protein; arrowhead indicates the catalytic tyrosine residue. (4.52 MB TIF) [file pone.0006859.s012.tif]
